# Polar Coding for Confidential Broadcasting

**DOI:** 10.3390/e22020149

**Published:** 2020-01-27

**Authors:** Jaume del Olmo Alòs, Javier Rodríguez Fonollosa

**Affiliations:** Departament de Teoria del Senyal i Communications, Universitat Politècnica de Catalunya, 08034 Barcelona, Spain; javier.fonollosa@upc.edu

**Keywords:** polar codes, information-theoretic security, wiretap broadcast channel, strong secrecy

## Abstract

A polar coding scheme is proposed for the Wiretap Broadcast Channel with two legitimate receivers and one eavesdropper. We consider a model in which the transmitter wishes to send the same private (non-confidential) message and the same confidential message reliably to two different legitimate receivers, and the confidential message must also be (strongly) secured from the eavesdropper. The coding scheme aims to use the optimal rate of randomness and does not make any assumption regarding the symmetry or degradedness of the channel. This paper extends previous work on polar codes for the wiretap channel by proposing a new chaining construction that allows to reliably and securely send the same confidential message to two different receivers. This construction introduces new dependencies between the random variables involved in the coding scheme that need to be considered in the secrecy analysis.

## 1. Introduction

Information-theoretic security over noisy channels was introduced by Wyner in [1], which characterized the secrecy-capacity of the degraded wiretap channel. Later, Csiszár and Körner in [2] generalized Wyner’s results to the general wiretap channel. In these settings, one transmitter wishes to reliably send one message to a legitimate receiver, while keeping it secret from an eavesdropper, where secrecy is defined based on a condition of some information-theoretic measure that is fully quantifiable. One of these measures is the *information leakage*, defined as the mutual information $I(W;Z^{n})$ between a uniformly distributed random message *W* and the channel observations $Z^{n}$ at the eavesdropper, *n* being the number of uses of the channel. Based on this measure, the most common secrecy conditions required to be satisfied by channel codes are the *weak secrecy*, which requires $\lim_{n\to \infty }\frac{1}{n}I\left(W;Z^{n}\right)=0$, and the *strong secrecy*, requiring $\lim_{n\to \infty }I\left(W;Z^{n}\right)=0$. Although the second notion of security is stronger, surprisingly both conditions result in the same secrecy-capacity [3].

In the last decade, information-theoretic security has been extended to a large variety of contexts, and polar codes have become increasingly popular in this area, due to their easily provable secrecy capacity achieving property. Polar codes were originally proposed by Arikan in [4] to achieve the capacity of binary-input, symmetric, and point-to-point channels under Successive Cancellation (SC) decoding. Secrecy capacity achieving polar codes for the binary symmetric degraded wiretap channel were introduced in [5,6], satisfying the weak and the strong secrecy condition, respectively. Recently, polar coding has been extended to the general wiretap channel in [7,8,9,10] and to different multiuser scenarios (for instance, see [11,12]). Indeed, [9,10] generalize their results providing polar codes for the broadcast channel with confidential messages.

This paper provides a polar coding scheme that allows to transmit *strongly* confidential common information to two legitimate receivers over the Wiretap Broadcast Channel (WTBC). Although [13] provided an obvious lower-bound on the secrecy-capacity of this model, no constructive polar coding scheme has already been proposed so far. Our polar coding scheme is based mainly on the one introduced by [10] for the broadcast channel with confidential messages. Therefore, the proposed polar coding scheme aims to use the optimal amount of randomness in the encoding. Moreover, in order to construct an explicit polar coding scheme that provides strong secrecy, the distribution induced by the encoder must be close in terms of the statistical distance to the original one considered for the code construction, and transmitter and legitimate receivers need to share a secret key of negligible size in terms of rate. Nevertheless, the particularization for the model proposed in this paper is not straightforward. Specifically, we propose a new chaining construction [14] (transmission will take place over several blocks) that is crucial to secretly transmit common information to different legitimate receivers. Indeed, this model generalizes, in part, the one described in [10], where the confidential message is intended only for one legitimate receiver, and the one in [15], which considers only the transmission of non-confidential messages intended for two different receivers. The proposed chaining introduces new bidirectional dependencies between encoding random variables of adjacent blocks that must be considered carefully in the secrecy analysis. Indeed, we need to make use of an additional secret key of negligible size in terms of rate that is privately shared between transmitter and legitimate receivers, which will be used to prove that dependencies between blocks can be broken and, therefore, the strong secrecy condition will be satisfied.

### 1.1. Notation

Throughout this paper, let [n]={1,…,n} for $n\in Z^{+}$, $a^{n}$ denotes a row vector (a(1),…,a(n)). We write $a^{1:j}$ for j∈[n] to denote the subvector (a(1),…,a(j)). Let A⊂[n], then we write a[A] to denote the sequence ${\left\{a\left(j\right)\right\}}_{j\in A}$, and we use $A^{C}$ to denote the set complement with respect to the universal set [n], that is, $A^{C}=\left[n\right]\setminus A$. If A denotes an event, then $A^{C}$ also denotes its complement. We use ln to denote the natural logarithm, whereas log denotes the logarithm base 2. Let *X* be a random variable taking values in X, and let $q_{x}$ and $p_{x}$ be two different distributions with support X, then $D(q_{x},p_{x})$ and $V(q_{x},p_{x})$ denote the Kullback–Leibler divergence and the total variation distance respectively. Finally, $h_{2}\left(p\right)$ denotes the binary entropy function, i.e., $h_{2}\left(p\right)=-p\log p-\left(1-p\right)\log\left(1-p\right)$.

### 1.2. Organization

The remainder of this paper is organized as follows. Section 2 introduces the channel model formally. In Section 3, the fundamental theorems of polar codes are revisited. Section 4 describes the proposed polar coding scheme, and Section 5 proves that this polar coding scheme achieves the best known inner-bound on the secrecy-capacity of this model. Finally, the concluding remarks are presented in Section 6.

## 2. Channel Model and Achievable Region

Formally, a WTBC $\left(X,p_{Y_{\left(1\right)}Y_{\left(2\right)}Z|X},Y_{\left(1\right)}\times Y_{\left(2\right)}\times Z\right)$ with 2 legitimate receivers and an external eavesdropper is characterized by the probability transition function $p_{Y_{\left(1\right)}Y_{\left(2\right)}Z|X}$, where X∈X denotes the channel input, $Y_{\left(k\right)}\in Y_{\left(k\right)}$ denotes the channel output corresponding to the legitimate Receiver k∈[1,2], and Z∈Z denotes the channel output corresponding to the eavesdropper. We consider a model, namely Common Information over the Wiretap Broadcast Channel (CI-WTBC), in which the transmitter wishes to send a private message *W* and a confidential message *S* to both legitimate receivers. A code $\left(\lceil 2^{nR_{W}}\rceil ,\lceil 2^{nR_{S}}\rceil ,\lceil 2^{nR_{R}}\rceil ,n\right)$ for the CI-WTBC consists of a private message set $W≜\left[1,\lceil 2^{nR_{W}}\rceil \right]$, a confidential message set $S≜\left[1,\lceil 2^{nR_{S}}\rceil \right]$, a randomization sequence set $R≜\left[1,\lceil 2^{nR_{R}}\rceil \right]$ (needed to confuse the eavesdropper about the confidential message *S*), an encoding function $f:W\times S\times R\to X^{n}$ that maps (w,s,r) to a codeword $x^{n}$, and two decoding functions $g_{\left(1\right)}$ and $g_{\left(2\right)}$ such that $g_{\left(k\right)}:Y_{\left(k\right)}^{n}\to W\times S$ (k∈[1,2]) maps the *k*-th legitimate receiver observations $y_{\left(k\right)}^{n}$ to the estimates $\left({\hat{w}}^{\left(k\right)},{\hat{s}}^{\left(k\right)}\right)$. The reliability condition to be satisfied by this code is measured in terms of the average probability of error and is given by

(1) $$\begin{array}{c} \lim_{n\to \infty }P\left[\left(W,S\right)\neq \left({\hat{W}}^{\left(k\right)},{\hat{S}}^{\left(k\right)}\right)\right]=0,\;k\in \left[1,2\right]. \end{array}$$

The *strong* secrecy condition is measured in terms of the information leakage and is given by

(2) $$\begin{array}{c} \lim_{n\to \infty }I\left(S;Z^{n}\right)=0. \end{array}$$

**Figure 1:**
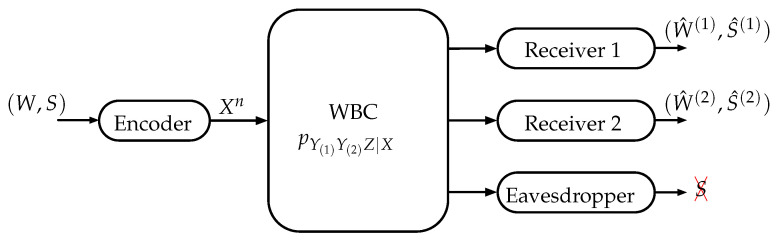
Channel model: CI-WTBC.

This model is graphically illustrated in Figure 1. A triple of rates $\left(R_{W},R_{S},R_{R}\right)\in R_{+}^{3}$ will be achievable for the CI-WTBC if there exists a sequence of $\left(\lceil 2^{nR_{W}}\rceil ,\lceil 2^{nR_{S}}\rceil ,\lceil 2^{nR_{R}}\rceil ,n\right)$ codes such that satisfy the reliability and secrecy conditions (1) and (2), respectively.

The achievable rate region is defined as the closure of the set of all achievable rate triples $\left(R_{W},R_{S},R_{R}\right)$. The following proposition defines an inner-bound on this region.

**Proposition** **1** (Adapted from [13,16])**.** *The region $R_{\mathrm{CI}-\mathrm{WTBC}}$ defined by the union over the triples of rates $\left(R_{W},R_{S},R_{R}\right)\in R_{+}^{3}$ satisfying*

$$\begin{array}{cc} R_{W}+R_{S} & \leq \min\left\{I\left(V;Y_{\left(1\right)}\right),I\left(V;Y_{\left(2\right)}\right)\right\},\\ R_{S} & \leq \min\left\{I\left(V;Y_{\left(1\right)}\right),I\left(V;Y_{\left(2\right)}\right)\right\}-I\left(V;Z\right),\\ R_{W}+R_{R} & \geq I(X;Z),\\ R_{R} & \geq I(X;Z|V), \end{array}$$

*where the union is taken over all distributions $p_{VX}$ such that $V-X-(Y_{\left(1\right)},Y_{\left(2\right)},Z)$ forms a Markov chain, defines an inner-bound on the achievable region of the CI-WTBC.*

In this model, the private message *W* introduces part of the randomness required to confuse the eavesdropper about the confidential message *S*, and the randomization sequence *R* denotes the additional randomness that is required for channel prefixing.

## 3. Review of Polar Codes

Let $\left(X\times Y,p_{XY}\right)$ be a Discrete Memoryless Source (DMS), where X∈{0,1} (Throughout this paper, we assume binary polarization. Nevertheless, an extension to *q*-ary alphabets is possible [10,17,18]) and Y∈Y. The polar transform over the *n*-sequence $X^{n}$, *n* being any power of 2, is defined as $U^{n}≜X^{n}G_{n}$, where $G_{n}≜{\left[\begin{array}{cc} 1 & 1\\ 1 & 0 \end{array}\right]}^{⊗n}$ is the source polarization matrix [19]. Since $G_{n}=G_{n}^{-1}$, then $X^{n}=U^{n}G_{n}$.

The polarization theorem for source coding with side information [19] (Th. 1) states that the polar transform extracts the randomness of $X^{n}$ in the sense that, as n→∞, the set of indices j∈[n] can be divided practically into two disjoint sets, namely $H_{X|Y}^{\left(n\right)}$ and $L_{X|Y}^{\left(n\right)}$, such that U(j) for $j\in H_{X|Y}^{\left(n\right)}$ is practically independent of $\left(U^{1:j-1},Y^{n}\right)$ and uniformly distributed, i.e., $H(U\left(j\right)|U^{1:j-1},Y^{n})\to 1$, and U(j) for $j\in L_{X|Y}^{\left(n\right)}$ is almost determined by $\left(U^{1:j-1},Y^{n}\right)$, i.e., $H(U\left(j\right)|U^{1:j-1},Y^{n})\to 0$. Formally, let

$$\begin{array}{cc} H_{X|Y}^{\left(n\right)} & ≜\left\{j\in \left[n\right]:H\left(U\left(j\right)|U^{1:j-1},Y^{n}\right)\geq 1-\delta_{n}\right\},\\ L_{X|Y}^{\left(n\right)} & ≜\left\{j\in \left[n\right]:H\left(U\left(j\right)|U^{1:j-1},Y^{n}\right)\leq \delta_{n}\right\}, \end{array}$$

where $\delta_{n}≜2^{-n^{\beta }}$ for some $\beta \in (0,\frac{1}{2})$. Then, by Lemma 4 of [10] we have $\lim_{n\to \infty }\frac{1}{n}\left|H_{X|Y}^{\left(n\right)}\right|=H\left(X|Y\right)$ and $\lim_{n\to \infty }\frac{1}{n}\left|L_{X|Y}^{\left(n\right)}\right|=1-H\left(X|Y\right)$, which imply that $\lim_{n\to \infty }\frac{1}{n}\left|{\left(H_{X|Y}^{\left(n\right)}\right)}^{C}\setminus L_{X|Y}^{\left(n\right)}\right|=0$, i.e., the number of elements that *have not been polarized* is asymptotically negligible in terms of rate. Furthermore, Th. 2 of [19] states that given $U[{\left(L_{X|Y}^{\left(n\right)}\right)}^{C}]$ and $Y^{n}$, $U[L_{X|Y}^{\left(n\right)}]$ can be reconstructed using SC decoding with error probability in $O(n\delta_{n})$. Alternatively, the previous sets can be defined based on the Bhattacharyya parameters ${\left\{Z\left(U\left(j\right)|U^{1:j-1},Y^{n}\right)\right\}}_{j=1}^{n}$ because both parameters *polarize* simultaneously Proposition 2 of [19]. It is worth mentioning that both the entropy terms and the Bhattacharyya parameters required to define these sets can be obtained deterministically from $p_{XY}$ and the algebraic properties of $G_{n}$ [20,21,22].

Similarly to $H_{X|Y}^{\left(n\right)}$ and $L_{X|Y}^{\left(n\right)}$, the sets $H_{X}^{\left(n\right)}$ and $L_{X}^{\left(n\right)}$ can be defined by considering that observations $Y^{n}$ are absent. A discrete memoryless channel $\left(X,p_{Y|X},Y\right)$ with some arbitrary $p_{X}$ can be seen as a DMS $\left(X\times Y,p_{X}p_{Y|X}\right)$. In channel polar coding, first we define $H_{X|Y}^{\left(n\right)}$, $L_{X|Y}^{\left(n\right)}$, $H_{X}^{\left(n\right)}$ and $L_{X}^{\left(n\right)}$ from the target distribution $p_{X}p_{Y|X}$ (*polar construction*). Then, based on the previous sets, the encoder somehow constructs (since the polar-based encoder will construct random variables that must approach the target distribution of the DMS, throughout this paper we use *tilde* above the random variables to emphazise this purpose) ${\tilde{U}}^{n}$ and applies the inverse polar transform ${\tilde{X}}^{n}={\tilde{U}}^{n}G_{n}$ with distribution ${\tilde{q}}_{X^{n}}$. Afterwards, the transmitter sends ${\tilde{X}}^{n}$ over the channel, which induces ${\tilde{Y}}^{n}∼{\tilde{q}}_{Y^{n}}$. If $V({\tilde{q}}_{X^{n}Y^{n}},p_{X^{n}Y^{n}})\to 0$, then the receiver can reliably reconstruct $\tilde{U}\left[L_{X|Y}^{\left(n\right)}\right]$ from ${\tilde{Y}}^{n}$ and $\tilde{U}\left[{\left(L_{X|Y}^{\left(n\right)}\right)}^{C}\right]$ by using SC decoding [23].

## 4. Polar Coding Scheme

Let $\left(V\times X\times Y_{\left(1\right)}\times Y_{\left(2\right)}\times Z,p_{VXY_{\left(1\right)}Y_{\left(2\right)}Z}\right)$ denote the DMS that represents the input (V,X) and output $\left(Y_{\left(1\right)},Y_{\left(2\right)},Z\right)$ random variables of the CI-WTBC, where |V|=|X|≜2. Without loss of generality, and to avoid the trivial case $R_{S}=0$ in Proposition 1, we assume that

(3) $$\begin{array}{c} H\left(V|Z\right)>H\left(V|Y_{\left(1\right)}\right)\geq H\left(V|Y_{\left(2\right)}\right). \end{array}$$

If $H\left(V|Y_{\left(1\right)}\right)<H\left(V|Y_{\left(2\right)}\right)$, one can simply exchange the role of $Y_{\left(1\right)}$ and $Y_{\left(2\right)}$ in the polar coding scheme described in Section 4. We propose a polar coding scheme that achieves the following rate triple,

(4) $$\begin{array}{c} \left(R_{W},R_{S},R_{R}\right)=\left(I\left(V;Z\right),I\left(V;Y_{\left(1\right)}\right)-I\left(V;Z\right),I\left(X;Z|V\right)\right), \end{array}$$

which corresponds to the one of the region in Proposition 1 such that the private and the confidential message rate are maximum and the amount of randomness is minimum.

For the input random variable *V*, we define the polar transform $A^{n}≜V^{n}G_{n}$ and the sets

(5) $$\begin{array}{cc} H_{V}^{\left(n\right)} & ≜\left\{j\in \left[1,n\right]:H\left(A\left(j\right)|A^{1:j-1}\right)\geq 1-\delta_{n}\right\}, \end{array}$$

(6) $$\begin{array}{cc} H_{V|Z}^{\left(n\right)} & ≜\left\{j\in \left[1,n\right]:H\left(A\left(j\right)|A^{1:j-1}Z^{n}\right)\geq 1-\delta_{n}\right\}, \end{array}$$

(7) $$\begin{array}{cc} L_{V|Y_{\left(k\right)}}^{\left(n\right)} & ≜\left\{j\in \left[1,n\right]:H\left(A\left(j\right)|A^{1:j-1}Y_{\left(k\right)}^{n}\right)\leq \delta_{n}\right\},\;k=1,2. \end{array}$$

For the input random variable *X*, we define $T^{n}≜X^{n}G_{n}$ and the associated sets

(8) $$\begin{array}{cc} H_{X|V}^{\left(n\right)} & ≜\left\{j\in \left[1,n\right]:H\left(T\left(j\right)|T^{1:j-1}V^{n}\right)\geq 1-\delta_{n}\right\}. \end{array}$$

(9) $$\begin{array}{cc} H_{X|VZ}^{\left(n\right)} & ≜\left\{j\in \left[1,n\right]:H\left(T\left(j\right)|T^{1:j-1}V^{n}Z^{n}\right)\geq 1-\delta_{n}\right\}. \end{array}$$

We have $p_{A^{n}T^{n}}\left(a^{n},t^{n}\right)=p_{V^{n}X^{n}}\left(a^{n}G_{n},t^{n}G_{n}\right)$, due to the invertibility of $G_{n}$, and we write

$$\begin{array}{cc} p_{A^{n}T^{n}}\left(a^{n},t^{n}\right)\; & =\left(\prod_{j=1}^{n}p_{A\left(j\right)|A^{1:j-1}}\left(a\left(j\right)|a^{1:j-1}\right)\right)\left(\prod_{j=1}^{n}p_{T\left(j\right)|T^{1:j-1}V^{n}}\left(t\left(j\right)|t^{1:j-1},a^{n}G_{n}\right)\right). \end{array}$$

Consider that the encoding takes place over *L* blocks indexed by i∈[1,L]. At the *i*-th block, the encoder will construct ${\tilde{A}}_{i}^{n}$, which will carry the private and the confidential messages intended for both legitimate receivers. Additionally, the encoder will store into ${\tilde{A}}_{i}^{n}$ some elements from ${\tilde{A}}_{i-1}^{n}$ (if i∈[2,L]) and ${\tilde{A}}_{i+1}^{n}$ (if i∈[1,L−1]), so that both legitimate receivers are able to reliably reconstruct ${\tilde{A}}_{1:L}^{n}$. Then, given ${\tilde{V}}_{i}^{n}={\tilde{A}}_{i}^{n}G_{n}$, the encoder will perform the polar-based channel prefixing to construct ${\tilde{T}}_{i}^{n}$. Finally, it will obtain ${\tilde{X}}_{i}^{n}={\tilde{T}}_{i}^{n}G_{n}$, which will be transmitted over the WTBC, inducing the channel output observations $\left({\tilde{Y}}_{(1),i}^{n},{\tilde{Y}}_{(2),i}^{n},{\tilde{Z}}_{i}^{n}\right)$.

Consider the construction of ${\tilde{A}}_{1:L}^{n}$. Besides, sets in (5)–(7), define the partition of $H_{V}^{\left(n\right)}$:

(10) $$\begin{array}{cc} G^{\left(n\right)} & ≜H_{V|Z}^{\left(n\right)}, \end{array}$$

(11) $$\begin{array}{cc} C^{\left(n\right)} & ≜H_{V}^{\left(n\right)}\cap {\left(H_{V|Z}^{\left(n\right)}\right)}^{C}. \end{array}$$

Moreover, we also define the following partition of the set $G^{\left(n\right)}$:

(12) $$\begin{array}{cc} G_{0}^{\left(n\right)} & ≜G^{\left(n\right)}\cap L_{V|Y_{\left(1\right)}}^{\left(n\right)}\cap L_{V|Y_{\left(2\right)}}^{\left(n\right)}, \end{array}$$

(13) $$\begin{array}{cc} G_{1}^{\left(n\right)} & ≜G^{\left(n\right)}\cap {\left(L_{V|Y_{\left(1\right)}}^{\left(n\right)}\right)}^{C}\cap L_{V|Y_{\left(2\right)}}^{\left(n\right)}, \end{array}$$

(14) $$\begin{array}{cc} G_{2}^{\left(n\right)} & ≜G^{\left(n\right)}\cap L_{V|Y_{\left(1\right)}}^{\left(n\right)}\cap {\left(L_{V|Y_{\left(2\right)}}^{\left(n\right)}\right)}^{C}, \end{array}$$

(15) $$\begin{array}{cc} G_{1,2}^{\left(n\right)} & ≜G^{\left(n\right)}\cap {\left(L_{V|Y_{\left(1\right)}}^{\left(n\right)}\right)}^{C}\cap {\left(L_{V|Y_{\left(2\right)}}^{\left(n\right)}\right)}^{C}, \end{array}$$

and the following partition of the set $C^{\left(n\right)}$:

(16) $$\begin{array}{cc} C_{0}^{\left(n\right)} & ≜C^{\left(n\right)}\cap L_{V|Y_{\left(1\right)}}^{\left(n\right)}\cap L_{V|Y_{\left(2\right)}}^{\left(n\right)}, \end{array}$$

(17) $$\begin{array}{cc} C_{1}^{\left(n\right)} & ≜C^{\left(n\right)}\cap {\left(L_{V|Y_{\left(1\right)}}^{\left(n\right)}\right)}^{C}\cap L_{V|Y_{\left(2\right)}}^{\left(n\right)}, \end{array}$$

(18) $$\begin{array}{cc} C_{2}^{\left(n\right)} & ≜C^{\left(n\right)}\cap L_{V|Y_{\left(1\right)}}^{\left(n\right)}\cap {\left(L_{V|Y_{\left(2\right)}}^{\left(n\right)}\right)}^{C}, \end{array}$$

(19) $$\begin{array}{cc} C_{1,2}^{\left(n\right)} & ≜C^{\left(n\right)}\cap {\left(L_{V|Y_{\left(1\right)}}^{\left(n\right)}\right)}^{C}\cap {\left(L_{V|Y_{\left(2\right)}}^{\left(n\right)}\right)}^{C}; \end{array}$$

**Figure 2:**
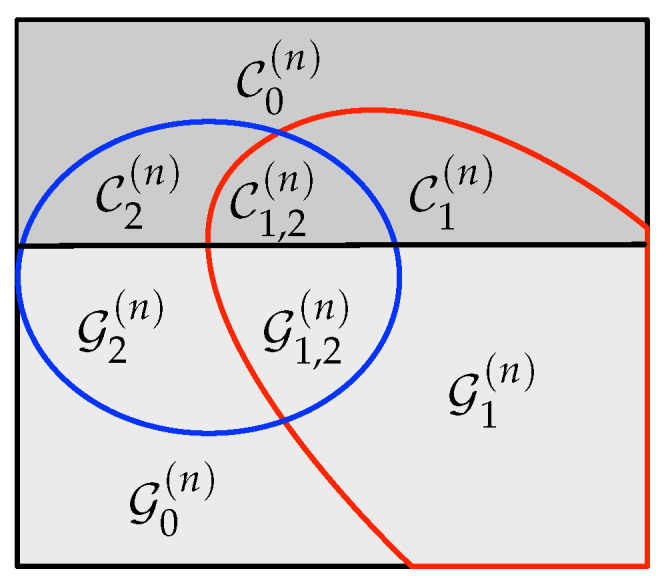
Graphical representation of the sets in (10)–(19). The indices inside the soft and dark gray area form $G^{\left(n\right)}$ and $C^{\left(n\right)}$ respectively. The indices that form $H_{V}^{\left(n\right)}\cap {\left(L_{V|Y_{\left(1\right)}}^{\left(n\right)}\right)}^{C}$ are those inside the red curve, while those inside the blue curve form $H_{V}^{\left(n\right)}\cap {\left(L_{V|Y_{\left(2\right)}}^{\left(n\right)}\right)}^{C}$.

These sets are graphically represented in Figure 2. Roughly speaking, $A[H_{V}^{\left(n\right)}]$ is the *nearly uniformly* distributed part of $A^{n}$. Thus, ${\tilde{A}}_{i}\left[H_{V}^{\left(n\right)}\right]$, i∈[1,L], is suitable for storing uniformly distributed random sequences. The sequence $A[H_{V|Z}^{\left(n\right)}]$ is *almost* independent of $Z^{n}$ and, hence, ${\tilde{A}}_{i}\left[G^{\left(n\right)}\right]$ is suitable for storing information to be secured from the eavesdropper, whereas ${\tilde{A}}_{i}\left[C^{\left(n\right)}\right]$ is not. Sets in (12)–(19) with subscript 1 (sets inside the red curve in Figure 2) form $H_{V}^{\left(n\right)}\cap {\left(L_{V|Y_{\left(1\right)}}^{\left(n\right)}\right)}^{C}$, while those with subscript 2 (sets inside the blue curve) form $H_{V}^{\left(n\right)}\cap {\left(L_{V|Y_{\left(2\right)}}^{\left(n\right)}\right)}^{C}$. From Th. 2 of [19,23], recall that ${\tilde{A}}_{i}\left[H_{V}^{\left(n\right)}\cap {\left(L_{V|Y_{\left(k\right)}}^{\left(n\right)}\right)}^{C}\right]$ is the nearly uniformly distributed part of the sequence ${\tilde{A}}_{i}^{n}$ required by legitimate Receiver *k* to reliably reconstruct the entire sequence by performing SC decoding.

For sufficiently large *n*, assumption (3) imposes the following restriction on the size of the previous sets:

(20) $$\begin{array}{c} |G_{1}^{\left(n\right)}|-|C_{2}^{\left(n\right)}|\geq |G_{2}^{\left(n\right)}|-|C_{1}^{\left(n\right)}|>|C_{1,2}^{\left(n\right)}|-|G_{0}^{\left(n\right)}|. \end{array}$$

The left-hand inequality in (20) holds from the fact that

$$\begin{array}{cc} \; & \left|C_{1}^{\left(n\right)}\cup G_{1}^{\left(n\right)}|-|C_{2}^{\left(n\right)}\cup G_{2}^{\left(n\right)}\right|\\ \; & \;=|H_{V}^{\left(n\right)}\cap {\left(L_{V|Y_{\left(1\right)}}^{\left(n\right)}\right)}^{C}\setminus H_{V}^{\left(n\right)}\cap {\left(L_{V|Y_{\left(2\right)}}^{\left(n\right)}\right)}^{C}|-|H_{V}^{\left(n\right)}\cap {\left(L_{V|Y_{\left(2\right)}}^{\left(n\right)}\right)}^{C}\setminus H_{V}^{\left(n\right)}\cap {\left(L_{V|Y_{\left(1\right)}}^{\left(n\right)}\right)}^{C}|\\ \; & \;=|H_{V}^{\left(n\right)}\cap {\left(L_{V|Y_{\left(1\right)}}^{\left(n\right)}\right)}^{C}|-|H_{V}^{\left(n\right)}\cap {\left(L_{V|Y_{\left(2\right)}}^{\left(n\right)}\right)}^{C}|\geq 0, \end{array}$$

where the positivity holds by Lemma 4 of [10] because, for any k∈[1,2], we have

$$\begin{array}{cc} \frac{1}{n}|H_{V}^{\left(n\right)}\cap {\left(L_{V|Y_{\left(k\right)}}^{\left(n\right)}\right)}^{C}|\; & =\frac{1}{n}|H_{V|Y_{\left(k\right)}}^{\left(n\right)}|+\frac{1}{n}|H_{V}^{\left(n\right)}\cap {\left(L_{V|Y_{\left(k\right)}}^{\left(n\right)}\right)}^{C}\setminus H_{V|Y_{\left(k\right)}}^{\left(n\right)}|\overset{n\to \infty }{\to }H\left(V|Y_{\left(k\right)}\right) \end{array}$$

Similarly, the right-hand inequality in (20) holds by Lemma 4 of [10] and the fact that

$$\begin{array}{cc} |G_{0}^{\left(n\right)}\cup G_{2}^{\left(n\right)}|-|C_{1}^{\left(n\right)}\cup C_{1,2}^{\left(n\right)}|\; & =|H_{V|Z}^{\left(n\right)}\setminus H_{V}^{\left(n\right)}\cap {\left(L_{V|Y_{\left(1\right)}}^{\left(n\right)}\right)}^{C}|-|H_{V}^{\left(n\right)}\cap {\left(L_{V|Y_{\left(1\right)}}^{\left(n\right)}\right)}^{C}\setminus H_{V|Z}^{\left(n\right)}|\\ \; & =|H_{V|Z}^{\left(n\right)}|-|H_{V}^{\left(n\right)}\cap {\left(L_{V|Y_{\left(1\right)}}^{\left(n\right)}\right)}^{C}|. \end{array}$$

Thus, according to (20), we must consider four cases:

- $|G_{1}^{\left(n\right)}\left|>\right|C_{2}^{\left(n\right)}|$, $|G_{2}^{\left(n\right)}\left|>\right|C_{1}^{\left(n\right)}|$ and $|G_{0}^{\left(n\right)}\left|\geq \right|C_{1,2}^{\left(n\right)}|$;
- $|G_{1}^{\left(n\right)}\left|>\right|C_{2}^{\left(n\right)}|$, $|G_{2}^{\left(n\right)}\left|>\right|C_{1}^{\left(n\right)}|$ and $|G_{0}^{\left(n\right)}\left|<\right|C_{1,2}^{\left(n\right)}|$;
- $|G_{1}^{\left(n\right)}\left|\geq \right|C_{2}^{\left(n\right)}|$, $|G_{2}^{\left(n\right)}\left|\leq \right|C_{1}^{\left(n\right)}|$ and $|G_{0}^{\left(n\right)}\left|>\right|C_{1,2}^{\left(n\right)}|$;
- $|G_{1}^{\left(n\right)}\left|<\right|C_{2}^{\left(n\right)}|$, $|G_{2}^{\left(n\right)}\left|<\right|C_{1}^{\left(n\right)}|$ and $|G_{0}^{\left(n\right)}\left|>\right|C_{1,2}^{\left(n\right)}|$.

### 4.1. General Polar-Based Encoding

The generic encoding process for all cases is summarized in Algorithm 1. For i∈[1,L], let $W_{i}$ be a uniformly distributed vector of length $|C^{\left(n\right)}|$ that represents the private message. The encoder forms ${\tilde{A}}_{i}\left[C^{\left(n\right)}\right]$ by simply storing $W_{i}$. Indeed, if i∈[1,L−1], notice that the encoder forms ${\tilde{A}}_{i+1}\left[C^{\left(n\right)}\right]$ before constructing ${\tilde{A}}_{i}^{n}$ entirely. From ${\tilde{A}}_{i}\left[C^{\left(n\right)}\right]$, i∈[1,L], we define

(21) $$\begin{array}{cc} \Psi_{i}^{\left(V\right)} & ≜{\tilde{A}}_{i}\left[C_{2}^{\left(n\right)}\right], \end{array}$$

(22) $$\begin{array}{cc} \Gamma_{i}^{\left(V\right)} & ≜{\tilde{A}}_{i}\left[C_{1,2}^{\left(n\right)}\right], \end{array}$$

(23) $$\begin{array}{cc} \Theta_{i}^{\left(V\right)} & ≜{\tilde{A}}_{i}\left[C_{1}^{\left(n\right)}\right]. \end{array}$$

Notice that $\left[\Psi_{i}^{\left(V\right)},\Gamma_{i}^{\left(V\right)}\right]={\tilde{A}}_{i}\left[C_{2}^{\left(n\right)}\cup C_{1,2}^{\left(n\right)}\right]$ is required by legitimate Receiver 2 to reliably estimate ${\tilde{A}}_{i}^{n}$ and, thus, the encoder will repeat $\left[\Psi_{i}^{\left(V\right)},\Gamma_{i}^{\left(V\right)}\right]$, if i∈[1,L−1], conveniently in ${\tilde{A}}_{i+1}\left[G^{\left(n\right)}\right]$ (the function form_A_G_ is responsible of the chaining construction and is described later). On the other hand, $\left[\Theta_{i}^{\left(V\right)},\Gamma_{i}^{\left(V\right)}\right]={\tilde{A}}_{i}\left[C_{1}^{\left(n\right)}\cup C_{1,2}^{\left(n\right)}\right]$ is required by legitimate Receiver 1. Nevertheless, in order to satisfy the strong secrecy condition in (2), $\left[\Theta_{i}^{\left(V\right)},\Gamma_{i}^{\left(V\right)}\right]$, i∈[2,L], is not repeated directly into ${\tilde{A}}_{i-1}\left[G^{\left(n\right)}\right]$, but the encoder copies instead ${\bar{\Theta }}_{i}^{\left(V\right)}$ and ${\bar{\Gamma }}_{i}^{\left(V\right)}$ obtained as follows. Let $\kappa_{\Theta }^{\left(V\right)}$ and $\kappa_{\Gamma }^{\left(V\right)}$ be uniformly distributed keys with length $|C_{1}^{\left(n\right)}|$ and $|C_{1,2}^{\left(n\right)}|$ respectively that are privately shared between transmitter and both legitimate receivers. For any i∈[2,L], we define the sequences

$$\begin{array}{cc} {\bar{\Theta }}_{i}^{\left(V\right)} & ≜{\tilde{A}}_{i}\left[C_{1}^{\left(n\right)}\right]⊕\kappa_{\Theta }^{\left(V\right)},\\ {\bar{\Gamma }}_{i}^{\left(V\right)} & ≜{\tilde{A}}_{i}\left[C_{1,2}^{\left(n\right)}\right]⊕\kappa_{\Gamma }^{\left(V\right)}. \end{array}$$

Since these secret keys are reused in all blocks, their size becomes negligible in terms of rate for *L* large enough. The need of these secret keys may not be obvious at this point, but a further discussion of this question can be found in Section 5.4. Indeed, they are required to prove independence between an eavesdropper’s observations of adjacent blocks (see Lemma 3), which is crucial to prove that the polar coding scheme satisfies the strong secrecy condition in (2).

The function form_A_G_ in Algorithm 1 constructs sequences ${\tilde{A}}_{1:L}\left[G^{\left(n\right)}\right]$ differently depending on which case, among cases A, B, C or D described before, characterizes the given CI-WTBC. This part of the encoding is described in detail in Section 4.2 and Algorithm 2.

Then, given ${\tilde{A}}_{i}\left[C^{\left(n\right)}\cup G^{\left(n\right)}\right]$, the encoder forms the remaining entries of ${\tilde{A}}_{i}^{n}$, i.e., ${\tilde{A}}_{i}\left[{\left(H_{V}^{\left(n\right)}\right)}^{C}\right]$, as follows. If $j\in L_{V}^{\left(n\right)}$, where $L_{V}^{\left(n\right)}≜\left\{j\in \left[1,n\right]:H\left(A\left(j\right)|A^{1:j-1}\right)\leq \delta_{n}\right\}$, it constructs ${\tilde{A}}_{i}\left(j\right)$ deterministically by using SC encoding [24], and only ${\tilde{A}}_{i}\left[{\left(H_{V}^{\left(n\right)}\right)}^{C}\setminus L_{V}^{\left(n\right)}\right]$ is constructed randomly.

Finally, given ${\tilde{V}}_{i}^{n}={\tilde{A}}_{i}^{n}G_{n}$, a randomization sequence $R_{i}$ and a uniformly distributed random sequence $\Lambda_{0}^{\left(V\right)}$, the encoder performs polar-based channel prefixing (function pb_ch_pref in Algorithm 1) to obtain ${\tilde{X}}_{i}^{n}$, which is transmitted over the WTBC inducing $\left({\tilde{Y}}_{(1),i}^{n},{\tilde{Y}}_{(2),i}^{n},{\tilde{Z}}_{i}^{n}\right)$. This part of the encoding is described in detail in Section 4.3.

Furthermore, the encoder obtains the sequence

$$\begin{array}{c} \Phi_{(k),i}^{\left(V\right)}≜{\tilde{A}}_{i}\left[{\left(H_{V}^{\left(n\right)}\right)}^{C}\cap {\left(L_{V|Y_{\left(k\right)}}^{\left(n\right)}\right)}^{C}\right] \end{array}$$

for any k∈[1,2] and i∈[1,L], which is required by legitimate Receiver *k* to reliably estimate ${\tilde{A}}_{i}^{n}$ entirely. Since $\Phi_{(k),i}^{\left(V\right)}$ is not *nearly uniform*, the encoder cannot make it available to the legitimate Receiver *k* by means of the chaining structure. Furthermore, the encoder obtains

$$\begin{array}{cc} \Upsilon_{\left(1\right)}^{\left(V\right)} & ≜{\tilde{A}}_{1}\left[H_{V}^{\left(n\right)}\cap {\left(L_{V|Y_{\left(1\right)}}^{\left(n\right)}\right)}^{C}\right],\\ \Upsilon_{\left(2\right)}^{\left(V\right)} & ≜{\tilde{A}}_{L}\left[H_{V}^{\left(n\right)}\cap {\left(L_{V|Y_{\left(2\right)}}^{\left(n\right)}\right)}^{C}\right]. \end{array}$$

The sequence $\Upsilon_{\left(k\right)}^{\left(V\right)}$ is required by legitimate Receiver k∈[1,2] to initialize the decoding process. Therefore, the transmitter additionally sends $\left(\Upsilon_{\left(k\right)}^{\left(V\right)},\Phi_{(k),i}^{\left(V\right)}\right)⊕\kappa_{{\Upsilon \Phi }_{\left(k\right)}}^{\left(V\right)}$ to legitimate Receiver *k*, where $\kappa_{{\Upsilon \Phi }_{\left(k\right)}}^{\left(V\right)}$ is a uniformly distributed key with size

$$\begin{array}{c} L|{\left(H_{V}^{\left(n\right)}\right)}^{C}\cap {\left(L_{V|Y_{\left(k\right)}}^{\left(n\right)}\right)}^{C}|+|H_{V}^{\left(n\right)}\cap {\left(L_{V|Y_{\left(k\right)}}^{\left(n\right)}\right)}^{C}| \end{array}$$

that is privately shared between transmitter and the corresponding receiver. In Section 5.1 we show that the length of $\kappa_{{\Upsilon \Phi }_{\left(1\right)}}^{\left(V\right)}$ and $\kappa_{{\Upsilon \Phi }_{\left(2\right)}}^{\left(V\right)}$ is asymptotically negligible in terms of rate.

**Algorithm 1** Generic encoding scheme**Require:** Private and confidential messages $W_{1:L}$ and $S_{1:L}$; randomization sequences $R_{1:L}$; random sequence $\Lambda_{0}^{\left(X\right)}$; and secret keys $\kappa_{\Theta }^{\left(V\right)}$, $\kappa_{\Gamma }^{\left(V\right)}$, $\kappa_{{\Upsilon \Phi }_{\left(1\right)}}^{\left(V\right)}$ and $\kappa_{{\Upsilon \Phi }_{\left(2\right)}}^{\left(V\right)}$.
 1:$\Psi_{0}^{\left(V\right)}$, $\Gamma_{0}^{\left(V\right)}$, $\Pi_{0}^{\left(V\right)}$, $\Lambda_{0}^{\left(V\right)}$, ${\bar{\Theta }}_{L+1}^{\left(V\right)}$, ${\bar{\Gamma }}_{L+1}^{\left(V\right)}\leftarrow ⌀$ 2:
${\tilde{A}}_{1}\left[C^{\left(n\right)}\right]\leftarrow W_{1}$
 3:
$\Psi_{1}^{\left(V\right)},\Gamma_{1}^{\left(V\right)}\leftarrow {\tilde{A}}_{1}\left[C^{\left(n\right)}\right]$
 4:**for**i=1 to *L* **do** 5:    **if** i≠L **then** 6:        ${\tilde{A}}_{i+1}\left[C^{\left(n\right)}\right]\leftarrow W_{i+1}$ 7:        $\Psi_{i+1}^{\left(V\right)},\Gamma_{i+1}^{\left(V\right)},{\bar{\Theta }}_{i+1}^{\left(V\right)},{\bar{\Gamma }}_{i+1}^{\left(V\right)}\leftarrow \;\left({\tilde{A}}_{i+1}\left[C^{\left(n\right)}\right],\kappa_{\Theta }^{\left(V\right)},\kappa_{\Gamma }^{\left(V\right)}\right)$ 8:    **end if** 9:    ${\tilde{A}}_{i}\left[G^{\left(n\right)}\right]$, $\Pi_{i}^{\left(V\right)}$, $\Lambda_{i}^{\left(V\right)}\leftarrow$form_A_G_$\left(i,S_{i},{\bar{\Theta }}_{i+1}^{\left(V\right)},{\bar{\Gamma }}_{i+1}^{\left(V\right)},\Psi_{i-1}^{\left(V\right)},\Gamma_{i-1}^{\left(V\right)},\Pi_{i-1}^{\left(V\right)},\Lambda_{i-1}^{\left(V\right)}\right)$10:    **if** i=1**then** $\Upsilon_{\left(1\right)}^{\left(V\right)}\leftarrow {\tilde{A}}_{1}\left[H_{V}^{\left(n\right)}\cap {\left(L_{V|Y_{\left(1\right)}}^{\left(n\right)}\right)}^{C}\right]$11:    **if** i=L**then** $\Upsilon_{\left(2\right)}^{\left(V\right)}\leftarrow {\tilde{A}}_{L}\left[H_{V}^{\left(n\right)}\cap {\left(L_{V|Y_{\left(2\right)}}^{\left(n\right)}\right)}^{C}\right]$12:    **for** $j\in {\left(H_{V}^{\left(n\right)}\right)}^{C}$ **do**13:        **if** $j\in {\left(H_{V}^{\left(n\right)}\right)}^{C}\setminus L_{V}^{\left(n\right)}$ **then**14:           ${\tilde{A}}_{i}\left(j\right)\leftarrow p_{A\left(j\right)|A^{1:j-1}}\left({\tilde{A}}_{i}\left(j\right)|{\tilde{A}}_{i}^{1:j-1}\right)$15:        **else if** $j\in L_{V}^{\left(n\right)}$ **then**16:           ${\tilde{A}}_{i}\left(j\right)\leftarrow {arg max}_{a\in V}p_{A\left(j\right)|A^{1:j-1}}\left({\tilde{a}}_{i}\left(j\right)|{\tilde{A}}_{i}^{1:j-1}\right)$17:        **end if**18:    **end for**19:    $\Phi_{(1),i}^{\left(V\right)}\leftarrow {\tilde{A}}_{i}\left[{\left(H_{V}^{\left(n\right)}\right)}^{C}\cap {\left(L_{V|Y_{\left(1\right)}}^{\left(n\right)}\right)}^{C}\right]$20:    $\Phi_{(2),i}^{\left(V\right)}\leftarrow {\tilde{A}}_{i}\left[{\left(H_{V}^{\left(n\right)}\right)}^{C}\cap {\left(L_{V|Y_{\left(2\right)}}^{\left(n\right)}\right)}^{C}\right]$21:    ${\tilde{X}}_{i}^{n},\Lambda_{i}^{\left(X\right)}\leftarrow \mathrm{pb\_ch\_pref}\left({\tilde{A}}_{i}^{n}G_{n},R_{i},\Lambda_{i-1}^{\left(X\right)}\right)$22:
**end for**
23:Send $\left(\Phi_{(k),i}^{\left(V\right)},\Upsilon_{\left(k\right)}^{\left(V\right)}\right)⊕\kappa_{{\Upsilon \Phi }_{\left(k\right)}}^{\left(V\right)}$ to Receiver k∈[1,2]24:
**return** ${\tilde{X}}_{1:L}^{n}$



**Algorithm 2** Function form_A_G_**Require:***i*, $S_{i}$, ${\bar{\Theta }}_{i+1}^{\left(V\right)}$, ${\bar{\Gamma }}_{i+1}^{\left(V\right)}$, $\Psi_{i-1}^{\left(V\right)}$, $\Gamma_{i-1}^{\left(V\right)}$, $\Pi_{i-1}^{\left(V\right)}$, $\Lambda_{i-1}^{\left(V\right)}$
 1:Define $R_{1}^{\left(n\right)}$, $R_{1}^{\prime (n)}$, $R_{2}^{\left(n\right)}$, $R_{2}^{\prime (n)}$, $R_{1,2}^{\left(n\right)}$, $R_{1,2}^{\prime (n)}$, $I^{\left(n\right)}$, $R_{S}^{\left(n\right)}$, $R_{\Lambda }^{\left(n\right)}$ (depending on the case) 2:
**if** i=1 **then** ${\tilde{A}}_{1}\left[I^{\left(n\right)}\cup G_{1}^{\left(n\right)}\cup G_{1,2}^{\left(n\right)}\right]\leftarrow S_{1}$
 3:
**if** i∈[2,L−1] **then** ${\tilde{A}}_{i}\left[I^{\left(n\right)}\right]\leftarrow S_{i}$
 4:
**if** i=L **then** ${\tilde{A}}_{L}\left[I^{\left(n\right)}\cup G_{2}^{\left(n\right)}\right]\leftarrow S_{L}$
 5:$\Psi_{1,i-1}^{\left(V\right)}$, $\Psi_{2,i-1}^{\left(V\right)}\leftarrow \Psi_{i-1}^{\left(V\right)}$ (depending on the case) 6:$\Gamma_{1,i-1}^{\left(V\right)}$, $\Gamma_{2,i-1}^{\left(V\right)}\leftarrow \Gamma_{i-1}^{\left(V\right)}$ (depending on the case) 7:${\bar{\Theta }}_{1,i+1}^{\left(V\right)}$, ${\bar{\Theta }}_{2,i+1}^{\left(V\right)}\leftarrow {\bar{\Theta }}_{i+1}^{\left(V\right)}$ (depending on the case) 8:${\bar{\Gamma }}_{1,i+1}^{\left(V\right)}$, ${\bar{\Gamma }}_{2,i+1}^{\left(V\right)}\leftarrow {\bar{\Gamma }}_{i+1}^{\left(V\right)}$ (depending on the case) 9:
${\tilde{A}}_{i}\left[R_{1,2}^{\left(n\right)}\right]\leftarrow \Gamma_{1,i-1}^{\left(V\right)}⊕{\bar{\Gamma }}_{1,i+1}^{\left(V\right)}$
10:
${\tilde{A}}_{i}\left[R_{1,2}^{\prime (n)}\right]\leftarrow \Psi_{2,i-1}^{\left(V\right)}⊕{\bar{\Theta }}_{2,i+1}^{\left(V\right)}$
11:
**if** i∈[1,L−1] **then**
12:    ${\tilde{A}}_{i}\left[R_{1}^{\left(n\right)}\right]\leftarrow {\bar{\Theta }}_{1,i+1}^{\left(V\right)}$13:    ${\tilde{A}}_{i}\left[R_{1}^{\prime (n)}\right]\leftarrow {\bar{\Gamma }}_{2,i+1}^{\left(V\right)}$14:
**end if**
15:
**if** i∈[2,L] **then**
16:    ${\tilde{A}}_{i}\left[R_{2}^{\left(n\right)}\right]\leftarrow \Psi_{1,i-1}^{\left(V\right)}$17:    ${\tilde{A}}_{i}\left[R_{2}^{\prime (n)}\right]\leftarrow \Gamma_{2,i-1}^{\left(V\right)}$18:    ${\tilde{A}}_{i}\left[R_{S}^{\left(n\right)}\right]\leftarrow \Pi_{i-1}^{\left(V\right)}$19:    ${\tilde{A}}_{i}\left[R_{\Lambda }^{\left(n\right)}\right]\leftarrow \Lambda_{i-1}^{\left(V\right)}$20:
**end if**
21:
$\Pi_{i}^{\left(V\right)}\leftarrow {\tilde{A}}_{i}\left[I^{\left(n\right)}\cap G_{2}^{\left(n\right)}\right]$
22:
$\Lambda_{i}^{\left(V\right)}\leftarrow {\tilde{A}}_{i}\left[R_{\Lambda }^{\left(n\right)}\right]$
23:**return** the sequences ${\tilde{A}}_{i}\left[G^{\left(n\right)}\right]$, $\Pi_{i}^{\left(V\right)}$ and $\Lambda_{i}^{\left(V\right)}$


### 4.2. Function form_A_G_

The function form_A_G_ encodes the confidential messages $S_{1:L}$ and builds the chaining construction. Based on the sets in (10)–(19), let $R_{1}^{\left(n\right)}\subseteq G_{0}^{\left(n\right)}\cup G_{2}^{\left(n\right)}$, $R_{1}^{\prime (n)}\subseteq G_{2}^{\left(n\right)}$, $R_{2}^{\left(n\right)}\subseteq G_{1}^{\left(n\right)}$, $R_{2}^{\prime (n)}\subseteq G_{1}^{\left(n\right)}$, $R_{1,2}^{\left(n\right)}\subseteq G_{0}^{\left(n\right)}$, $R_{1,2}^{\prime (n)}\subseteq G_{0}^{\left(n\right)}$, $I^{\left(n\right)}\subseteq G_{0}^{\left(n\right)}\cup G_{2}^{\left(n\right)}$, $R_{S}^{\left(n\right)}\subseteq G_{1}^{\left(n\right)}$ and $R_{\Lambda }^{\left(n\right)}\subseteq G_{1}^{\left(n\right)}$ form an additional partition of $G^{\left(n\right)}$. The definition of $R_{1}^{\left(n\right)}$, $R_{1}^{\prime (n)}$, $R_{2}^{\left(n\right)}$, $R_{2}^{\prime (n)}$, $R_{1,2}^{\left(n\right)}$ and $R_{1,2}^{\prime (n)}$ will depend on the particular case (among A to D), while

(24) $$\begin{array}{cc} I^{\left(n\right)} & ≜\left(G_{0}^{\left(n\right)}\cup G_{2}^{\left(n\right)}\right)\setminus \left(R_{1}^{\left(n\right)}\cup R_{1}^{\prime (n)}\cup R_{1,2}^{\left(n\right)}\cup R_{1,2}^{\prime (n)}\right), \end{array}$$

(25) $$\begin{array}{cc} R_{S}^{\left(n\right)} & ≜\mathrm{any}\;\mathrm{subset}\;\mathrm{of}\;G_{1}^{\left(n\right)}\setminus \left(R_{2}^{\left(n\right)}\cup R_{2}^{\prime (n)}\right)\;\mathrm{with}\;\mathrm{size}\;|I^{\left(n\right)}\cap G_{2}^{\left(n\right)}|, \end{array}$$

(26) $$\begin{array}{cc} R_{\Lambda }^{\left(n\right)} & ≜G_{1,2}^{\left(n\right)}\cup \left(G_{1}^{\left(n\right)}\setminus \left(R_{2}^{\left(n\right)}\cup R_{2}^{\prime (n)}\cup R_{S}^{\left(n\right)}\right)\right). \end{array}$$

For i∈[1,L], let $S_{i}$ denote a uniformly distributed vector that represents the confidential message. The message $S_{1}$ has size $\left|I^{\left(n\right)}\cup G_{1}^{\left(n\right)}\cup G_{1,2}^{\left(n\right)}\right|$; for i∈[2,L−1], $S_{i}$ has size $\left|I^{\left(n\right)}\right|$; and $S_{L}$ has size $\left|I^{\left(n\right)}\cup G_{2}^{\left(n\right)}\right|$. Furthermore, for i∈[1,L], we write $\Psi_{i}^{\left(V\right)}≜\left[\Psi_{1,i}^{\left(V\right)},\Psi_{2,i}^{\left(V\right)}\right]$, $\Gamma_{i}^{\left(V\right)}≜\left[\Gamma_{1,i}^{\left(V\right)},\Gamma_{2,i}^{\left(V\right)}\right]$, ${\bar{\Theta }}_{i}^{\left(V\right)}≜\left[{\bar{\Theta }}_{1,i}^{\left(V\right)},{\bar{\Theta }}_{2,i}^{\left(V\right)}\right]$ and ${\bar{\Gamma }}_{i}^{\left(V\right)}≜\left[{\bar{\Gamma }}_{1,i}^{\left(V\right)},{\bar{\Gamma }}_{2,i}^{\left(V\right)}\right]$, where we define $\Psi_{p,i}$, $\Gamma_{p,i}$, ${\bar{\Theta }}_{p,i}$ and ${\bar{\Gamma }}_{p,i}$, for any p∈[1,2], accordingly in each case.

This function, which is used in Case A to Case D, is described in Algorithm 2.

#### 4.2.1. Case A

In this case, recall that $|G_{1}^{\left(n\right)}\left|>\right|C_{2}^{\left(n\right)}|$, $|G_{2}^{\left(n\right)}\left|>\right|C_{1}^{\left(n\right)}|$ and $|G_{0}^{\left(n\right)}\left|\geq \right|C_{1,2}^{\left(n\right)}|$. We define

(27) $$\begin{array}{cc} R_{1}^{\left(n\right)} & ≜\mathrm{any}\;\mathrm{subset}\;\mathrm{of}\;G_{2}^{\left(n\right)}\;\mathrm{with}\;\mathrm{size}\;|C_{1}^{\left(n\right)}|, \end{array}$$

(28) $$\begin{array}{cc} R_{2}^{\left(n\right)} & ≜\mathrm{any}\;\mathrm{subset}\;\mathrm{of}\;G_{1}^{\left(n\right)}\;\mathrm{with}\;\mathrm{size}\;|C_{2}^{\left(n\right)}|, \end{array}$$

(29) $$\begin{array}{cc} R_{1,2}^{\left(n\right)} & ≜\mathrm{any}\;\mathrm{subset}\;\mathrm{of}\;G_{0}^{\left(n\right)}\;\mathrm{with}\;\mathrm{size}\;|C_{1,2}^{\left(n\right)}|, \end{array}$$

and $R_{1}^{\prime (n)}=R_{2}^{\prime (n)}=R_{1,2}^{\prime (n)}≜\emptyset$. By the assumption of Case A, it is clear that $R_{1}^{\left(n\right)}$, $R_{2}^{\left(n\right)}$ and $R_{1,2}^{\left(n\right)}$ exist. Furthermore, by (20), the set $I^{\left(n\right)}$ exists, and so will $R_{S}^{\left(n\right)}$ because

$$\begin{array}{cc} \left|G_{1}^{\left(n\right)}\setminus \left(R_{2}^{\left(n\right)}\cup R_{2}^{\prime (n)}\right)|-|I^{\left(n\right)}\cap G_{2}^{\left(n\right)}\right| & =|G_{1}^{\left(n\right)}\setminus \left(R_{2}^{\left(n\right)}\cup R_{2}^{\prime (n)}\right)|-|\left(G_{2}^{\left(n\right)}\setminus R_{1}^{\left(n\right)}\cup R_{1}^{\prime (n)}\right)|\\ \; & =|G_{1}^{\left(n\right)}|-|C_{2}^{\left(n\right)}|-|G_{2}^{\left(n\right)}|-|C_{1}^{\left(n\right)}|\geq 0. \end{array}$$

**Figure 3:**
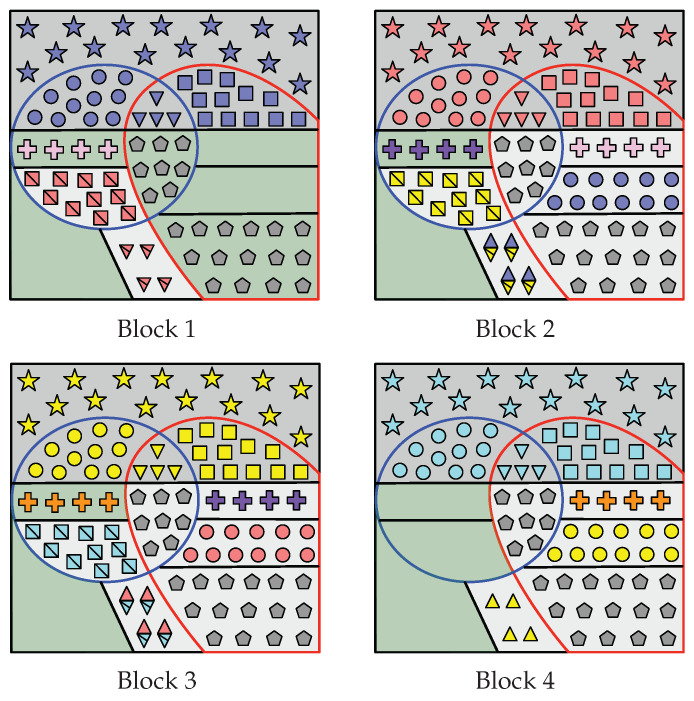
For Case A, graphical representation of the encoding that leads to the construction of ${\tilde{A}}_{1:L}\left[H_{V}^{\left(n\right)}\right]$ when L=4. Consider the Block 2: $R_{1}^{\left(n\right)}$, $R_{2}^{\left(n\right)}$, $R_{1,2}^{\left(n\right)}$, $R_{S}^{\left(n\right)}$ and $R_{\Lambda }^{\left(n\right)}$ are those areas filled with yellow squares, blue circles, blue and yellow diamonds, pink crosses, and gray pentagons, respectively, and the set $I^{\left(n\right)}$ is the green filled area. At Block i∈[1,L], $W_{i}$ is represented by symbols of the same color (e.g., red symbols at Block 2), and $\Theta_{i}^{\left(V\right)}$, $\Psi_{i}^{\left(V\right)}$ and $\Gamma_{i}^{\left(V\right)}$ are represented by squares, circles and triangles respectively. Furthermore, ${\bar{\Theta }}_{i}^{\left(V\right)}$ and ${\bar{\Gamma }}_{i}^{\left(V\right)}$ are denoted by squares and triangles, respectively, with a line through them. At Block i∈[2,L−1], the diamonds denote $\Gamma_{1,i-1}^{\left(V\right)}⊕{\bar{\Gamma }}_{1,i+1}^{\left(V\right)}$. In Block i∈[1,L], $S_{i}$ is stored into those entries whose indices belong to the green area. For i∈[1,L−1], $\Pi_{i}^{\left(V\right)}$ is denoted by crosses (e.g., purple crosses at Block 2), and is repeated in ${\tilde{A}}_{i+1}\left[R_{S}^{\left(n\right)}\right]$. The sequence $\Lambda_{1}^{\left(V\right)}$ is represented by gray pentagons and is replicated in all blocks. The sequences $\Upsilon_{\left(1\right)}^{\left(V\right)}$ and $\Upsilon_{\left(2\right)}^{\left(V\right)}$ are those entries inside the red at Block 1 and the blue curve at Block *L*, respectively.

These sets that form the partition of $G^{\left(n\right)}$ in Case A can be seen in Figure 3, which also displays the encoding process that aims to construct ${\tilde{A}}_{1:L}\left[H_{V}^{\left(n\right)}\right]={\tilde{A}}_{1:L}\left[C^{\left(n\right)}\cup G^{\left(n\right)}\right]$.

For i∈[1,L], we define $\Psi_{1,i}^{\left(V\right)}≜\Psi_{i}^{\left(V\right)}$, $\Gamma_{1,i}^{\left(V\right)}≜\Gamma_{i}^{\left(V\right)}$, ${\bar{\Theta }}_{1,i}^{\left(V\right)}≜{\bar{\Theta }}_{i}^{\left(V\right)}$, ${\bar{\Gamma }}_{1,i}^{\left(V\right)}≜{\bar{\Gamma }}_{i}^{\left(V\right)}$ and, therefore, we have $\Psi_{2,i}^{\left(V\right)}=\Gamma_{2,i}^{\left(V\right)}={\bar{\Theta }}_{2,i}^{\left(V\right)}={\bar{\Gamma }}_{2,i}^{\left(V\right)}≜⌀$.

From (18), we have $C_{2}^{\left(n\right)}\subseteq L_{V|Y_{\left(1\right)}}^{\left(n\right)}\setminus L_{V|Y_{\left(2\right)}}^{\left(n\right)}$. Thus, the sequence $\Psi_{1,i-1}^{\left(V\right)}={\tilde{A}}_{i-1}\left[C_{2}^{\left(n\right)}\right]$ is needed by legitimate Receiver 2 to reliably reconstruct ${\tilde{A}}_{i-1}^{n}$, but can be reliably inferred by legitimate Receiver 1 given ${\tilde{A}}_{i-1}\left[{\left(L_{V|Y_{\left(1\right)}}^{\left(n\right)}\right)}^{C}\right]$. Hence, according to Algorithm 2, the encoder repeats the entire sequence $\Psi_{1,i-1}^{\left(V\right)}$ in ${\tilde{A}}_{i}\left[R_{2}^{\left(n\right)}]\subseteq {\tilde{A}}_{i}[L_{V|Y_{\left(2\right)}}^{\left(n\right)}\setminus L_{V|Y_{\left(1\right)}}^{\left(n\right)}\right]$.

Similarly, from (17), we have $C_{1}^{\left(n\right)}\subseteq L_{V|Y_{\left(2\right)}}^{\left(n\right)}\setminus L_{V|Y_{\left(1\right)}}^{\left(n\right)}$. Thus, $\Theta_{1,i+1}^{\left(V\right)}={\tilde{A}}_{i+1}\left[C_{1}^{\left(n\right)}\right]$ is needed by Receiver 1 to form ${\tilde{A}}_{i+1}^{n}$ but can be inferred by Receiver 2 given ${\tilde{A}}_{i+1}\left[{\left(L_{V|Y_{\left(2\right)}}^{\left(n\right)}\right)}^{C}\right]$. Hence, the encoder repeats the sequence ${\bar{\Theta }}_{1,i+1}^{\left(V\right)}$ in ${\tilde{A}}_{i}\left[R_{1}^{\left(n\right)}\right]\subseteq {\tilde{A}}_{i}\left[L_{V|Y_{\left(1\right)}}^{\left(n\right)}\setminus L_{V|Y_{\left(2\right)}}^{\left(n\right)}\right]$.

Finally, from (19), $C_{1,2}^{\left(n\right)}\subseteq {\left(L_{V|Y_{\left(2\right)}}^{\left(n\right)}\right)}^{C}\cap {\left(L_{V|Y_{\left(1\right)}}^{\left(n\right)}\right)}^{C}$. Thus, sequences $\Gamma_{1,i-1}^{\left(V\right)}={\tilde{A}}_{i-1}\left[C_{1,2}^{\left(n\right)}\right]$ and $\Gamma_{1,i+1}^{\left(V\right)}={\tilde{A}}_{i+1}\left[C_{1,2}^{\left(n\right)}\right]$ are needed by both receivers to form ${\tilde{A}}_{i-1}^{n}$ and ${\tilde{A}}_{i+1}^{n}$ respectively. Hence, the encoder repeats $\Gamma_{1,i-1}^{\left(V\right)}$ and ${\bar{\Gamma }}_{1,i+1}^{\left(V\right)}$ in ${\tilde{A}}_{i}\left[R_{1,2}^{\left(n\right)}\right]\subseteq {\tilde{A}}_{i}\left[L_{V|Y_{\left(1\right)}}^{\left(n\right)}\cap L_{V|Y_{\left(2\right)}}^{\left(n\right)}\right]$. Indeed, both sequences are repeated in the same entries of ${\tilde{A}}_{i}\left[G_{0}^{\left(n\right)}\right]$ by performing $\Gamma_{1,i-1}^{\left(V\right)}⊕{\bar{\Gamma }}_{1,i+1}^{\left(V\right)}$. Since $\Gamma_{1,0}^{\left(V\right)}={\bar{\Gamma }}_{1,L+1}^{\left(V\right)}=⌀$, only ${\bar{\Gamma }}_{1,2}^{\left(V\right)}$ is repeated at Block 1 and $\Gamma_{1,L-1}^{\left(V\right)}$ at Block *L*.

Moreover, part of secret message $S_{i}$, i∈[1,L], is stored into some entries of ${\tilde{A}}_{i}^{n}$ whose indices belong to $G_{2}^{\left(n\right)}$. Thus, in any Block i∈[2,L], the encoder repeats

$$\begin{array}{c} \Pi_{i-1}^{\left(V\right)}≜{\tilde{A}}_{i-1}\left[I^{\left(n\right)}\cap G_{2}^{\left(n\right)}\right] \end{array}$$

in ${\tilde{A}}_{i}\left[R_{S}^{\left(n\right)}\right]\subseteq {\tilde{A}}_{i}\left[L_{V|Y_{\left(2\right)}}^{\left(n\right)}\setminus L_{V|Y_{\left(1\right)}}^{\left(n\right)}\right]$. Furthermore, it repeats

$$\begin{array}{c} \Lambda_{i-1}^{\left(V\right)}≜{\tilde{A}}_{i-1}\left[R_{\Lambda }^{\left(n\right)}\right] \end{array}$$

in ${\tilde{A}}_{i}\left[R_{\Lambda }^{\left(n\right)}\right]$. Hence, notice that $\Lambda_{1}^{\left(V\right)}$ is replicated in all blocks.

#### 4.2.2. Case B

In this case, $\left|G_{1}^{\left(n\right)}|>|C_{2}^{\left(n\right)}\right|$, $\left|G_{2}^{\left(n\right)}|>|C_{1}^{\left(n\right)}\right|$ and $\left|G_{0}^{\left(n\right)}|<|C_{1,2}^{\left(n\right)}\right|$. We define $R_{1}^{\left(n\right)}$ and $R_{2}^{\left(n\right)}$ as in (27) and (28) respectively, and $R_{1,2}^{\prime (n)}≜\emptyset$. Now, since $\left|G_{0}^{\left(n\right)}|<|C_{1,2}^{\left(n\right)}\right|$, only a part of $\Gamma_{i-1}^{\left(V\right)}$ and ${\bar{\Gamma }}_{i+1}^{\left(V\right)}$, i∈[1,L], can be repeated in ${\tilde{A}}_{i}\left[G_{0}^{\left(n\right)}\right]$. Thus, we define $R_{1,2}^{\left(n\right)}≜G_{0}^{\left(n\right)}$ and

(30) $$\begin{array}{cc} R_{1}^{\prime (n)} & ≜\mathrm{any}\;\mathrm{subset}\;\mathrm{of}\;G_{2}^{\left(n\right)}\setminus R_{1}^{\left(n\right)}\;\mathrm{with}\;\mathrm{size}\;|C_{1,2}^{\left(n\right)}|-|G_{0}^{\left(n\right)}|, \end{array}$$

(31) $$\begin{array}{cc} R_{2}^{\prime (n)} & ≜\mathrm{any}\;\mathrm{subset}\;\mathrm{of}\;G_{1}^{\left(n\right)}\setminus R_{2}^{\left(n\right)}\;\mathrm{with}\;\mathrm{size}\;|C_{1,2}^{\left(n\right)}|-|G_{0}^{\left(n\right)}|. \end{array}$$

Obviously, $R_{1,2}^{\left(n\right)}$ exists and, by the assumption of Case B, so do $R_{1}^{\left(n\right)}$ and $R_{2}^{\left(n\right)}$. By (20), $R_{1}^{\prime (n)}$ exists and so does $I^{\left(n\right)}$. Indeed, since $G_{0}^{\left(n\right)}\setminus R_{1,2}^{\left(n\right)}=\emptyset$, then $I^{\left(n\right)}\subseteq G_{2}^{\left(n\right)}$. Again, by the property in (20), $R_{2}^{\prime (n)}$ exists and so does $R_{S}^{\left(n\right)}$ because

$$\begin{array}{cc} \; & \left|G_{1}^{\left(n\right)}\setminus \left(R_{2}^{\left(n\right)}\cup R_{2}^{\prime (n)}\right)|-|\left(G_{2}^{\left(n\right)}\setminus R_{1}^{\left(n\right)}\cup R_{1}^{\prime (n)}\right)\right|\\ \; & \;=|G_{1}^{\left(n\right)}|-|C_{2}^{\left(n\right)}|-\left(|C_{1,2}^{\left(n\right)}|-|G_{0}^{\left(n\right)}|\right)-\left(|G_{2}^{\left(n\right)}|-|C_{1}^{\left(n\right)}|-\left(|C_{1,2}^{\left(n\right)}|-|G_{0}^{\left(n\right)}|\right)\right)\\ \; & \;=|G_{1}^{\left(n\right)}|-|C_{2}^{\left(n\right)}|-|G_{2}^{\left(n\right)}|+|C_{1}^{\left(n\right)}|\geq 0. \end{array}$$

**Figure 4:**
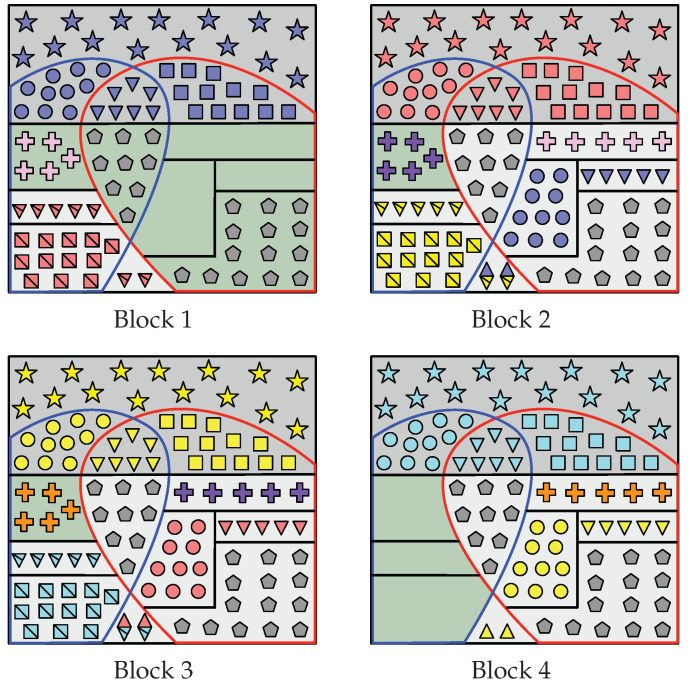
For Case B, graphical representation of the encoding that leads to the construction of ${\tilde{A}}_{1:L}\left[H_{V}^{\left(n\right)}\right]$ when L=4. Consider the Block 2: the sets $R_{1}^{\left(n\right)}$, $R_{1}^{\prime (n)}$, $R_{2}^{\left(n\right)}$, $R_{2}^{\prime (n)}$, $R_{1,2}^{\left(n\right)}$, $R_{S}^{\left(n\right)}$ and $R_{\Lambda }^{\left(n\right)}$ are those areas filled with yellow squares, yellow triangles, blue circles, blue triangles, blue and yellow diamonds, pink crosses, and gray pentagons, respectively, and $I^{\left(n\right)}$ is the green filled area with purple crosses. At Block i∈[1,L], $W_{i}$ is represented by symbols of the same color (e.g., red symbols at Block 2), and $\Theta_{i}^{\left(V\right)}$, $\Psi_{i}^{\left(V\right)}$ and $\Gamma_{i}^{\left(V\right)}$ are represented by squares, circles, and triangles, respectively. Furthermore, ${\bar{\Theta }}_{i}^{\left(V\right)}$ and ${\bar{\Gamma }}_{i}^{\left(V\right)}$ are denoted by squares and triangles, respectively, with a line through them. At Block i∈[2,L−1], the diamonds denote $\Gamma_{1,i-1}^{\left(V\right)}⊕{\bar{\Gamma }}_{1,i+1}^{\left(V\right)}$. In Block i∈[1,L], $S_{i}$ is stored into those entries whose indices belong to the green area. For i∈[2,L−1], $\Pi_{i}^{\left(V\right)}=S_{i}$ and, therefore, $S_{i}$ is repeated entirely into ${\tilde{A}}_{i+1}\left[R_{S}^{\left(n\right)}\right]$. The sequence $\Lambda_{1}^{\left(V\right)}$ from $S_{1}$ is represented by gray pentagons and is repeated in all blocks. The sequences $\Upsilon_{\left(1\right)}^{\left(V\right)}$ and $\Upsilon_{\left(2\right)}^{\left(V\right)}$ are the entries inside the red curve at Block 1 and the blue curve at Block *L*, respectively.

Indeed, since $I^{\left(n\right)}\subseteq G_{2}^{\left(n\right)}$, notice that $\left|R_{S}^{\left(n\right)}|=|I^{\left(n\right)}\right|$. These sets that form the partition of $G^{\left(n\right)}$ in Case B can be seen in Figure 4, which also displays the encoding process that aims to construct ${\tilde{A}}_{1:L}\left[H_{V}^{\left(n\right)}\right]={\tilde{A}}_{1:L}\left[C^{\left(n\right)}\cup G^{\left(n\right)}\right]$.

In this case, for any i∈[1,L], $\Psi_{1,i}^{\left(V\right)}≜\Psi_{i}^{\left(V\right)}$, ${\bar{\Theta }}_{1,i}^{\left(V\right)}≜{\bar{\Theta }}_{i}^{\left(V\right)}$ and $\Psi_{2,i}^{\left(V\right)}={\bar{\Theta }}_{2,i}^{\left(V\right)}≜⌀$; and we define $\Gamma_{1,i}^{\left(V\right)}$ and ${\bar{\Gamma }}_{1,i}^{\left(V\right)}$ as any part of $\Gamma_{i}^{\left(V\right)}$ and ${\bar{\Gamma }}_{i}^{\left(V\right)}$, respectively, with size $\left|G_{0}^{\left(n\right)}\right|$, and $\Gamma_{2,i}^{\left(V\right)}$ and ${\bar{\Gamma }}_{2,i}^{\left(V\right)}$ as the remaining parts with size $\left|C_{1,2}^{\left(n\right)}|-|G_{0}^{\left(n\right)}\right|$. Now, the encoder copies $\Gamma_{1,i-1}^{\left(V\right)}⊕{\bar{\Gamma }}_{1,i+1}^{\left(V\right)}$ into ${\tilde{A}}_{i}\left[R_{1,2}^{\left(n\right)}\right]$, and $\Gamma_{2,i-1}^{\left(V\right)}$ and ${\bar{\Gamma }}_{2,i+1}^{\left(V\right)}$ into ${\tilde{A}}_{i}\left[R_{2}^{\prime (n)}\right]$ and ${\tilde{A}}_{i}\left[R_{1}^{\prime (n)}\right]$ respectively. Moreover, since $I^{\left(n\right)}\subseteq G_{2}^{\left(n\right)}$, notice that $\Pi_{i}^{\left(V\right)}=S_{i}$ for any i∈[2,L−1].

#### 4.2.3. Case C

In this case, recall that $\left|G_{1}^{\left(n\right)}|\geq |C_{2}^{\left(n\right)}\right|$, $\left|G_{2}^{\left(n\right)}|\leq |C_{1}^{\left(n\right)}\right|$ and $\left|G_{0}^{\left(n\right)}|>|C_{1,2}^{\left(n\right)}\right|$. Hence, we define $R_{2}^{\left(n\right)}$ and $R_{1,2}^{\left(n\right)}$ as in (28) and (29) respectively, and $R_{1}^{\prime (n)}=R_{2}^{\prime (n)}=R_{1,2}^{\prime (n)}≜\emptyset$. On the other hand, since $\left|G_{2}^{\left(n\right)}|\leq |C_{1}^{\left(n\right)}\right|$, now for i∈[1,L−1] only a part of ${\bar{\Theta }}_{i+1}^{\left(V\right)}$ can be repeated entirely in ${\tilde{A}}_{i}\left[G_{2}^{\left(n\right)}\right]$. Consequently, we define

(32) $$\begin{array}{c} R_{1}^{\left(n\right)}≜\mathrm{the}\;\mathrm{union}\;\mathrm{of}\;G_{2}^{\left(n\right)}\;\mathrm{with}\;\mathrm{any}\;\mathrm{subset}\;\mathrm{of}\;G_{0}^{\left(n\right)}\setminus R_{1,2}^{\left(n\right)}\;\mathrm{with}\;\mathrm{size}\;|C_{1}^{\left(n\right)}|-|G_{2}^{\left(n\right)}|. \end{array}$$

**Figure 5:**
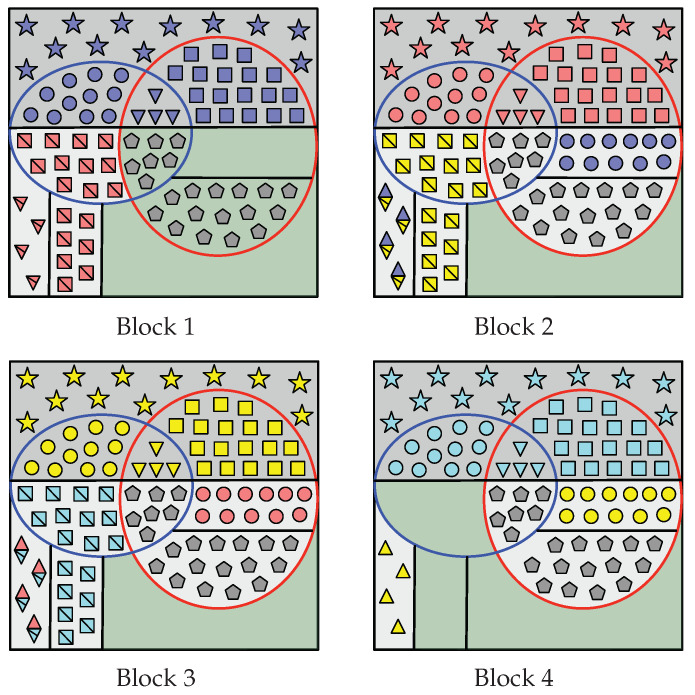
For Case C, graphical representation of the encoding that leads to the construction of ${\tilde{A}}_{1:L}\left[H_{V}^{\left(n\right)}\right]$ when L=4. Consider the Bloc 2: $R_{1}^{\left(n\right)}$, $R_{2}^{\left(n\right)}$, $R_{1,2}^{\left(n\right)}$ and $R_{\Lambda }^{\left(n\right)}$ are those areas filled with yellow squares, blue circles, blue and yellow diamonds, and gray pentagons, respectively, and $I^{\left(n\right)}$ is the green filled area. At Block i∈[1,L], $W_{i}$ is represented by symbols of the same color (e.g., red symbols at Block 2), and $\Theta_{i}^{\left(V\right)}$, $\Psi_{i}^{\left(V\right)}$ and $\Gamma_{i}^{\left(V\right)}$ are represented by squares, circles, and triangles, respectively. Furthermore, ${\bar{\Theta }}_{i}^{\left(V\right)}$ and ${\bar{\Gamma }}_{i}^{\left(V\right)}$ are denoted by squares and triangles, respectively, with a line through them. At Block i∈[2,L−1], the diamonds denote $\Gamma_{1,i-1}^{\left(V\right)}⊕{\bar{\Gamma }}_{1,i+1}^{\left(V\right)}$. For i∈[1,L], $S_{i}$ is stored into those entries belonging to the green area. The sequence $\Lambda_{1}^{\left(V\right)}$ is represented by gray pentagons and is repeated in all blocks. The sequences $\Upsilon_{\left(1\right)}^{\left(V\right)}$ and $\Upsilon_{\left(2\right)}^{\left(V\right)}$ are the entries inside the red curve at Block 1 and the blue curve at Block *L*, respectively.

It is clear that $R_{2}^{\left(n\right)}$ and $R_{1,2}^{\left(n\right)}$ exist. By (20), $R_{1}^{\left(n\right)}$ also exists and so does $I^{\left(n\right)}$. Since $R_{1}^{\left(n\right)}⊇G_{2}^{\left(n\right)}$, then $I^{\left(n\right)}\cap G_{2}^{\left(n\right)}=\emptyset$ and $R_{S}^{\left(n\right)}=\emptyset$. These sets that form $G^{\left(n\right)}$ are represented in Figure 5, which also displays the part of the encoding that aims to construct ${\tilde{A}}_{1:L}\left[H_{V}^{\left(n\right)}\right]$.

In this case, for i∈[1,L], we define $\Psi_{1,i}^{\left(V\right)}≜\Psi_{i}^{\left(V\right)}$, $\Gamma_{1,i}^{\left(V\right)}≜\Gamma_{i}^{\left(V\right)}$, ${\bar{\Theta }}_{1,i}^{\left(V\right)}≜{\bar{\Theta }}_{i}^{\left(V\right)}$, ${\bar{\Gamma }}_{1,i}^{\left(V\right)}≜{\bar{\Gamma }}_{i}^{\left(V\right)}$, and $\Psi_{2,i}^{\left(V\right)}=\Gamma_{2,i}^{\left(V\right)}={\bar{\Theta }}_{2,i}^{\left(V\right)}={\bar{\Gamma }}_{2,i}^{\left(V\right)}≜⌀$. Moreover, note that $\Pi_{i}^{\left(V\right)}=⌀$ because $I^{\left(n\right)}\cap G_{2}^{\left(n\right)}=\emptyset$.

#### 4.2.4. Case D

**Figure 6:**
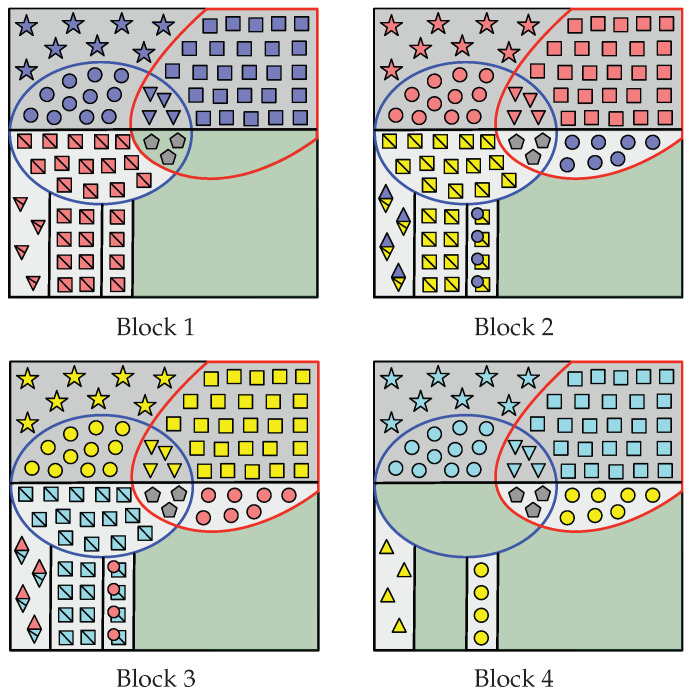
For Case D, graphical representation of the encoding that leads to the construction of ${\tilde{A}}_{1:L}\left[H_{V}^{\left(n\right)}\right]$ when L=4. Consider the Block 2: $R_{1}^{\left(n\right)}$, $R_{2}^{\left(n\right)}$, $R_{1,2}^{\left(n\right)}$, $R_{1,2}^{\prime (n)}$ and $R_{\Lambda }^{\left(n\right)}$ are those areas filled with yellow squares, blue circles, blue and yellow diamonds, yellow squares overlapped by blue circles, and gray pentagons, respectively, and the set $I^{\left(n\right)}$ is the green filled area. At Block i∈[1,L], $W_{i}$ is represented by symbols of the same color (e.g., red symbols at Block 2), and $\Theta_{i}^{\left(V\right)}$, $\Psi_{i}^{\left(V\right)}$ and $\Gamma_{i}^{\left(V\right)}$ are represented by squares, circles, and triangles, respectively. Furthermore, ${\bar{\Theta }}_{i}^{\left(V\right)}$ and ${\bar{\Gamma }}_{i}^{\left(V\right)}$ are denoted by squares and triangles, respectively, with a line through them. At Block i∈[2,L−1], $\Gamma_{1,i-1}^{\left(V\right)}⊕{\bar{\Gamma }}_{1,i+1}^{\left(V\right)}$ is represented by diamonds, and $\Psi_{2,i-1}^{\left(V\right)}⊕{\bar{\Theta }}_{2,i+1}^{\left(V\right)}$ by squares overlapped by circles. At Block i∈[1,L], $S_{i}$ is stored into those entries that belong to the green area. Sequence $\Lambda_{1}^{\left(V\right)}$ is denoted by gray pentagons and is repeated in all blocks. Sequences $\Upsilon_{\left(1\right)}^{\left(V\right)}$ and $\Upsilon_{\left(2\right)}^{\left(V\right)}$ are the entries inside the red curve at Block 1 and the blue curve at Block *L*, respectively.

In this case, recall that $\left|G_{1}^{\left(n\right)}|<|C_{2}^{\left(n\right)}\right|$, $\left|G_{2}^{\left(n\right)}|<|C_{1}^{\left(n\right)}\right|$ and $\left|G_{0}^{\left(n\right)}|>|C_{1,2}^{\left(n\right)}\right|$. The sets that form the partition of $G^{\left(n\right)}$ in Case D are defined below and can be seen in Figure 6, which also displays the encoding process that aims to construct of ${\tilde{A}}_{1:L}\left[H_{V}^{\left(n\right)}\right]$.

As in Case A and Case C, since $\left|G_{0}^{\left(n\right)}|>|C_{1,2}^{\left(n\right)}\right|$ then we define the set $R_{1,2}^{\left(n\right)}$ as in (29) and $R_{1}^{\prime (n)}=R_{2}^{\prime (n)}≜\emptyset$. On the other hand, since $|G_{1}^{\left(n\right)}\left|<\right|C_{2}^{\left(n\right)}|$, now for i∈[2,L] only a part of $\Psi_{i-1}^{\left(V\right)}$ can be repeated entirely in ${\tilde{A}}_{i}\left[G_{1}^{\left(n\right)}\right]$. Consequently, we define $R_{2}^{\left(n\right)}≜G_{1}^{\left(n\right)}$ and

(33) $$\begin{array}{c} R_{1,2}^{\prime (n)}≜\mathrm{any}\;\mathrm{subset}\;\mathrm{of}\;G_{0}^{\left(n\right)}\setminus R_{1,2}^{\left(n\right)}\;\mathrm{with}\;\mathrm{size}\;|C_{2}^{\left(n\right)}|-|G_{1}^{\left(n\right)}|. \end{array}$$

By (20), it is clear that $R_{1,2}^{\prime (n)}$ exists. Now, despite $\left|G_{2}^{\left(n\right)}|<|C_{1}^{\left(n\right)}\right|$ as in Case C, the set $R_{1}^{\left(n\right)}$ is not defined as in (32), but

(34) $$\begin{array}{cc} R_{1}^{\left(n\right)} & ≜\mathrm{the}\;\mathrm{union}\;\mathrm{of}\;G_{2}^{\left(n\right)}\;\mathrm{with}\;\mathrm{any}\;\mathrm{subset}\;\\ \; & \;\;\;\mathrm{of}\;G_{0}^{\left(n\right)}\setminus \left(R_{1,2}^{\left(n\right)}\cup R_{1,2}^{\prime (n)}\right)\;\mathrm{with}\;\mathrm{size}\;|C_{1}^{\left(n\right)}|-|G_{2}^{\left(n\right)}|-\left(|C_{2}^{\left(n\right)}|-|G_{1}^{\left(n\right)}|\right), \end{array}$$

which exists because, by the assumption in (20), we have

$$\begin{array}{cc} \; & \left|G_{0}^{\left(n\right)}\setminus \left(R_{1,2}^{\left(n\right)}\cup R_{1,2}^{\prime (n)}\right)|-|R_{1}^{\left(n\right)}\right|\\ \; & \;=|G_{0}^{\left(n\right)}|-|C_{1,2}^{\left(n\right)}|-|C_{2}^{\left(n\right)}|+|G_{1}^{\left(n\right)}|-\left(|C_{1}^{\left(n\right)}|-|G_{2}^{\left(n\right)}|-|C_{2}^{\left(n\right)}|+|G_{1}^{\left(n\right)}|\right)\\ \; & \;=|G_{0}^{\left(n\right)}|-|C_{1,2}^{\left(n\right)}|-|C_{1}^{\left(n\right)}|+|G_{2}^{\left(n\right)}|\geq 0. \end{array}$$

In this case, for i∈[1,L], we set $\Gamma_{1,i}^{\left(V\right)}≜\Gamma_{i}^{\left(V\right)}$, ${\bar{\Gamma }}_{1,i}^{\left(V\right)}≜{\bar{\Gamma }}_{i}^{\left(V\right)}$ and ${\bar{\Gamma }}_{2,i}^{\left(V\right)}=\Gamma_{2,i}^{\left(V\right)}≜⌀$. Furthermore, we define $\Psi_{1,i}^{\left(V\right)}$ as any part of $\Psi_{i}^{\left(V\right)}$ with size $\left|G_{1}^{\left(n\right)}\right|$, and $\Psi_{2,i}^{\left(V\right)}$ as the remaining part with size $\left|C_{2}^{\left(n\right)}|-|G_{1}^{\left(n\right)}\right|$. Lastly, we define ${\bar{\Theta }}_{1,i}^{\left(V\right)}$ as any part ${\bar{\Theta }}_{i}^{\left(V\right)}$ with size $|C_{1}^{\left(n\right)}|-\left(|C_{2}^{\left(n\right)}|-|G_{1}^{\left(n\right)}|\right)$, and ${\bar{\Theta }}_{2,i}^{\left(V\right)}$ as the remaining part with size $\left|C_{2}^{\left(n\right)}|-|G_{1}^{\left(n\right)}\right|$.

Thus, according to Algorithm 2, instead of repeating $\Psi_{2,i-1}^{\left(V\right)}$, that is, the part of $\Psi_{i-1}^{\left(V\right)}$ that does not fit in ${\tilde{A}}_{i}^{n}\left[G_{1}^{\left(n\right)}\right]$, in a specific part of ${\tilde{A}}_{i}\left[G_{0}^{\left(n\right)}\right]$, the encoder stores $\Psi_{2,i-1}^{\left(V\right)}⊕{\bar{\Theta }}_{2,i+1}^{\left(V\right)}$ into ${\tilde{A}}_{i}\left[R_{1,2}^{\prime (n)}\right]\subseteq {\tilde{A}}_{i}\left[G_{0}^{\left(n\right)}\right]$, where ${\bar{\Theta }}_{2,i+1}^{\left(V\right)}$ denotes part of those elements of ${\bar{\Theta }}_{i+1}^{\left(V\right)}$ that do not fit in ${\tilde{A}}_{i}\left[G_{2}^{\left(n\right)}\right]$. Furthermore, as in Case C, since $I^{\left(n\right)}\cap G_{2}^{\left(n\right)}=\emptyset$, we have $\Pi_{i}^{\left(V\right)}=⌀$.

### 4.3. Channel Prefixing

For i∈[1,L], let $R_{i}$ be a uniformly distributed vector of length $\left|H_{X|V}^{\left(n\right)}\setminus H_{X|VZ}^{\left(n\right)}\right|$ that represents the randomization sequence. Furthermore, let $\Lambda_{0}^{\left(X\right)}$ be a uniformly distributed random sequence of size $\left|H_{X|VZ}^{\left(n\right)}\right|$. The channel prefixing aims to construct ${\tilde{X}}_{i}^{n}={\tilde{T}}_{i}^{n}G_{n}$ and is summarized in Algorithm 3.

**Algorithm 3** Function pb_ch_pref**Require:**${\tilde{V}}_{i}^{n}$, $R_{i}$, $\Lambda_{i-1}^{\left(X\right)}$
 1:
${\tilde{T}}_{i}\left[H_{X|VZ}^{\left(n\right)}\right]\leftarrow \Lambda_{i-1}^{\left(X\right)}$
 2:
${\tilde{T}}_{i}\left[H_{X|V}^{\left(n\right)}\setminus H_{X|VZ}^{\left(n\right)}\right]\leftarrow R_{i}$
 3:
**for** $j\in {\left(H_{X|V}^{\left(n\right)}\right)}^{C}$ **do**
 4:    **if** $j\in {\left(H_{X|V}^{\left(n\right)}\right)}^{C}\setminus L_{X|V}^{\left(n\right)}$ **then** 5:        ${\tilde{T}}_{i}\left(j\right)\leftarrow p_{T\left(j\right)|T^{1:j-1}V^{n}}\left({\tilde{T}}_{i}\left(j\right)|{\tilde{T}}_{i}^{1:j-1}{\tilde{V}}_{i}^{n}\right)$ 6:    **else if** $j\in L_{X|V}^{\left(n\right)}$ **then** 7:        ${\tilde{T}}_{i}\left(j\right)\leftarrow {arg max}_{t\in X}p_{T\left(j\right)|T^{1:j-1}V^{n}}\left(t|{\tilde{T}}_{i}^{1:j-1}{\tilde{V}}_{i}^{n}\right)$ 8:    **end if** 9:
**end for**
10:
${\tilde{X}}_{i}^{n}\leftarrow {\tilde{T}}_{i}^{n}G_{n}$
11:
$\Lambda_{i}^{\left(X\right)}\leftarrow {\tilde{T}}_{i}\left[H_{X|V}^{\left(n\right)}\setminus H_{X|VZ}^{\left(n\right)}\right]$
12:**return**${\tilde{X}}_{i}^{n}$ and $\Lambda_{i}^{\left(X\right)}$


Notice that the sequence $\Lambda_{0}^{\left(X\right)}$ is copied in ${\tilde{T}}_{i}\left[H_{X|VZ}^{\left(n\right)}\right]$ at any Block i∈[1,L], while $R_{i}$ is stored into ${\tilde{T}}_{i}\left[H_{X|V}^{\left(n\right)}\setminus H_{X|VZ}^{\left(n\right)}\right]$. After forming ${\tilde{T}}_{i}\left[H_{X|V}^{\left(n\right)}\right]$, and given the sequence ${\tilde{V}}_{i}^{n}≜{\tilde{A}}_{i}^{n}G_{n}$, the encoder forms the remaining entries of ${\tilde{T}}_{i}^{n}$, that is, ${\tilde{T}}_{i}\left[{\left(H_{X|V}^{\left(n\right)}\right)}^{C}\right]$ as follows. If $j\in L_{X|V}^{\left(n\right)}$, where $L_{V|X}^{\left(n\right)}≜\left\{j\in \left[1,n\right]:H\left(T\left(j\right)|T^{1:j-1}V^{n}\right)\leq \delta_{n}\right\}$, it constructs ${\tilde{T}}_{i}\left(j\right)$ deterministically by using SC encoding [24]. Otherwise, if $j\in (H_{X|V}^{\left(n\right)}{)}^{C}\setminus L_{X|V}^{\left(n\right)}$, the encoder randomly draws ${\tilde{T}}_{i}\left(j\right)$ from distribution $p_{T\left(j\right)|T^{1:j-1}V^{n}}$.

### 4.4. Decoding

Consider that $\left(\Upsilon_{\left(k\right)}^{\left(V\right)},\Phi_{(k),1:L}^{\left(V\right)}\right)$, for all k∈[1,2], is available to the *k*-th legitimate receiver. In the decoding process, both legitimate receivers form the estimates ${\hat{A}}_{1:L}^{n}$ of ${\tilde{A}}_{1:L}^{n}$ and then obtain the messages $\left({\hat{W}}_{1:L},{\hat{S}}_{1:L}\right)$.

#### 4.4.1. Legitimate Receiver 1

This receiver forms the estimates ${\hat{A}}_{1:L}^{n}$ by going forward, i.e., from ${\hat{A}}_{1}^{n}$ to ${\hat{A}}_{L}^{n}$, and this process is summarized in Algorithm 4.

**Algorithm 4** Decoding at legitimate Receiver 1**Require:**$\Upsilon_{\left(1\right)}^{\left(V\right)}$, $\Phi_{(1),1:L}^{\left(V\right)}$, $\kappa_{\Theta }^{\left(V\right)}$ and $\kappa_{\Gamma }^{\left(V\right)}$, and ${\tilde{Y}}_{(1),1:L}^{n}$.
 1:
${\hat{A}}_{1}^{n}\leftarrow \left(\Upsilon_{\left(1\right)}^{\left(V\right)},\Phi_{(1),1}^{\left(V\right)},{\tilde{Y}}_{(1),1}^{n}\right)$
 2:
${\hat{\Lambda }}_{2:L}^{\left(V\right)}\leftarrow {\hat{A}}_{1}\left[R_{\Lambda }^{\left(n\right)}\right]$
 3:**for**i=1 to L−1 **do** 4:    ${\hat{\Psi }}_{i}^{\left(V\right)}\leftarrow {\hat{A}}_{i}\left[C_{2}^{\left(n\right)}\right]$ 5:    ${\hat{\Gamma }}_{i}^{\left(V\right)}\leftarrow {\hat{A}}_{i}\left[C_{1,2}^{\left(n\right)}\right]$ 6:    ${\hat{\bar{\Theta }}}_{i+1}^{\left(V\right)}\leftarrow \left({\hat{A}}_{i}\left[R_{1}^{\left(n\right)}\right],{\hat{A}}_{i}\left[R_{1,2}^{\prime (n)}\right]⊕{\hat{\Psi }}_{2,i-1}^{\left(V\right)}\right)$ 7:    ${\hat{\Theta }}_{i+1}^{\left(V\right)}\leftarrow {\hat{\bar{\Theta }}}_{i+1}^{\left(V\right)}⊕\kappa_{\Theta }^{\left(V\right)}$ 8:    ${\hat{\bar{\Gamma }}}_{i+1}^{\left(V\right)}\leftarrow \left({\hat{A}}_{i}\left[R_{1,2}^{\left(n\right)}\right]⊕{\hat{\Gamma }}_{1,i-1}^{\left(V\right)},{\hat{A}}_{i}\left[R_{1}^{\prime (n)}\right]\right)$ 9:    ${\hat{\Gamma }}_{i+1}^{\left(V\right)}\leftarrow {\hat{\bar{\Gamma }}}_{i+1}^{\left(V\right)}⊕\kappa_{\Gamma }^{\left(V\right)}$10:    ${\hat{\Pi }}_{i}^{\left(V\right)}\leftarrow {\hat{A}}_{i}\left[I^{\left(n\right)}\cap G_{2}^{\left(n\right)}\right]$11:    ${\hat{\Upsilon }}_{(1),i+1}^{\prime (V)}\leftarrow \left({\hat{\Psi }}_{1,i}^{\left(V\right)},{\hat{\Gamma }}_{2,i}^{\left(V\right)},{\hat{\Theta }}_{i+1}^{\left(V\right)},{\hat{\Gamma }}_{i+1}^{\left(V\right)},{\hat{\Pi }}_{i}^{\left(V\right)},{\hat{\Lambda }}_{i}^{\left(V\right)}\right)$12:    ${\hat{A}}_{i+1}^{n}\leftarrow \left({\hat{\Upsilon }}_{(1),i+1}^{\prime (V)},\Phi_{(1),i+1}^{\left(V\right)},{\tilde{Y}}_{(1),i+1}^{n}\right)$13:
**end for**



In all cases (among Case A to Case D), Receiver 1 constructs ${\hat{A}}_{1}^{n}$ as follows. Given $\Upsilon_{\left(1\right)}^{\left(V\right)}$ (all the elements inside the red curve at Block 1 in Figure 3, Figure 4, Figure 5 and Figure 6) and $\Phi_{(1),1}^{\left(V\right)}$, notice that Receiver 1 knows ${\tilde{A}}_{1}\left[{\left(L_{V|Y_{\left(1\right)}}^{\left(n\right)}\right)}^{C}\right]$. Therefore, from $\left(\Upsilon_{\left(1\right)}^{\left(V\right)},\Phi_{(1),1}^{\left(V\right)}\right)$ and channel observations ${\tilde{Y}}_{(1),1}^{n}$, Receiver 1 performs SC decoding to form ${\hat{A}}_{1}^{n}$. Moreover, since $\Lambda_{1}^{\left(V\right)}$ has been replicated in all blocks, legitimate Receiver 1 obtains ${\hat{\Lambda }}_{2:L}^{\left(V\right)}={\hat{A}}_{1}\left[R_{\Lambda }^{\left(n\right)}\right]$ (gray pentagons in all blocks).

For i∈[1,L−1], consider the construction of ${\hat{A}}_{i+1}^{n}$. First, since ${\hat{A}}_{1:i}^{n}$ have already been estimated, from ${\hat{A}}_{i}^{n}$ Receiver 1 obtains ${\hat{\Psi }}_{i}^{\left(V\right)}={\hat{A}}_{i}\left[C_{2}^{\left(n\right)}\right]$ (e.g., red circles at Block 2 in Figure 3, Figure 4, Figure 5 and Figure 6) and ${\hat{\Gamma }}_{i}^{\left(V\right)}={\hat{A}}_{i}\left[C_{1,2}^{\left(n\right)}\right]$ (red triangles).

Furthermore, from ${\hat{A}}_{i}^{n}$, Receiver 1 obtains ${\hat{\Theta }}_{i+1}^{\left(V\right)}$ as follows. At Block 1, in all cases it gets ${\hat{\bar{\Theta }}}_{2}^{\left(V\right)}={\tilde{A}}_{1}\left[R_{1}^{\left(n\right)}\cup R_{1,2}^{\prime (n)}\right]$ (all the red squares with a line through them at Block 1 in Figure 3, Figure 4, Figure 5 and Figure 6). At Block i∈[2,L−1], we distinguish two situations:

- In Case D, Receiver 1 gets ${\hat{\bar{\Theta }}}_{1,i+1}^{\left(V\right)}={\hat{A}}_{i}\left[R_{1}^{\left(V\right)}\right]$ (e.g., yellow squares with a line through them at Block 2 in Figure 6) and ${\hat{\Psi }}_{2,i-1}^{\left(V\right)}⊕{\hat{\bar{\Theta }}}_{2,i+1}^{\left(V\right)}={\hat{A}}_{i}\left[R_{1,2}^{\prime (V)}\right]$ (yellow squares with a line through them overlapped by blue circles). Since ${\hat{\Psi }}_{2,i-1}^{\left(V\right)}\subset {\hat{A}}_{i-1}^{n}$ (blue circles) has already been estimated, Receiver 1 obtains ${\hat{\bar{\Theta }}}_{2,i+1}^{\left(V\right)}={\hat{\Psi }}_{2,i-1}^{\left(V\right)}⊕{\hat{A}}_{i}\left[R_{1,2}^{\prime (V)}\right]$ (yellow squares with a line through them).
- Otherwise, in other cases, Receiver 1 obtains ${\hat{\bar{\Theta }}}_{i+1}^{\left(V\right)}={\hat{A}}_{i}\left[R_{1}^{\left(n\right)}\right]$ directly (yellow squares with a line through them at Block 2 in Figure 3, Figure 4 and Figure 5).

Then, given ${\hat{\bar{\Theta }}}_{i+1}^{\left(V\right)}=\left[{\hat{\bar{\Theta }}}_{1,i+1}^{\left(V\right)},{\hat{\bar{\Theta }}}_{2,i+1}^{\left(V\right)}\right]$, in all cases Receiver 1 recovers ${\hat{\Theta }}_{i+1}^{\left(V\right)}={\hat{\bar{\Theta }}}_{i+1}^{\left(V\right)}⊕\kappa_{\Theta }^{\left(V\right)}$.

From ${\hat{A}}_{i}^{n}$, Receiver 1 also obtains ${\hat{\Gamma }}_{i+1}^{\left(V\right)}$ as follows. At Block 1, in all cases it gets ${\hat{\bar{\Gamma }}}_{2}^{\left(V\right)}={\tilde{A}}_{1}\left[R_{1,2}^{\left(n\right)}\cup R_{1}^{\prime (n)}\right]$ directly (e.g., all red triangles with a line through them at Block 1 in Figure 3, Figure 4, Figure 5 and Figure 6). At Block i∈[2,L−1], in all cases it obtains ${\hat{\Gamma }}_{1,i-1}^{\left(V\right)}⊕{\hat{\bar{\Gamma }}}_{1,i+1}^{\left(V\right)}={\hat{A}}_{i}\left[R_{1,2}^{\left(n\right)}\right]$ (e.g., blue and yellow diamonds with a line through them at Block 2). Since ${\hat{\Gamma }}_{1,i-1}^{\left(V\right)}\subset {\hat{A}}_{i-1}^{n}$ (blue triangles) has already been estimated, Receiver 1 obtains ${\hat{\bar{\Gamma }}}_{1,i+1}^{\left(V\right)}={\hat{A}}_{i}\left[R_{1,2}^{\left(n\right)}\right]⊕{\hat{\Gamma }}_{1,i-1}^{\left(V\right)}$ (yellow triangles with a line through them). Only in Case B, Receiver 1 obtains ${\hat{\bar{\Gamma }}}_{2,i+1}^{\left(V\right)}={\hat{A}}_{i}\left[R_{1}^{\prime (n)}\right]$ (remaining yellow triangles with a line through them at Block 2 in Figure 4). Then, given ${\hat{\bar{\Gamma }}}_{i+1}^{\left(V\right)}=\left[{\hat{\bar{\Gamma }}}_{1,i+1}^{\left(V\right)},{\hat{\bar{\Gamma }}}_{2,i+1}^{\left(V\right)}\right]$, in all cases Receiver 1 recovers ${\hat{\Gamma }}_{i+1}^{\left(V\right)}={\hat{\bar{\Gamma }}}_{i+1}^{\left(V\right)}⊕\kappa_{\Gamma }^{\left(V\right)}$.

Lastly, only in Case A and Case B, Receiver 1 obtains ${\hat{\Pi }}_{i}^{\left(V\right)}={\hat{A}}_{i}\left[I^{\left(n\right)}\cap G_{2}^{\left(n\right)}\right]$ (e.g., purple crosses at Block 2 in Figure 3 and Figure 4).

Finally, define the sequence ${\hat{\Upsilon }}_{(1),i+1}^{\prime (V)}≜\left[{\hat{\Psi }}_{1,i}^{\left(V\right)},{\hat{\Gamma }}_{2,i}^{\left(V\right)},{\hat{\Theta }}_{i+1}^{\left(V\right)},{\hat{\Gamma }}_{i+1}^{\left(V\right)},{\hat{\Pi }}_{i}^{\left(V\right)},{\hat{\Lambda }}_{i}^{\left(V\right)}\right]$. Notice that ${\hat{\Upsilon }}_{(1),i+1}^{\prime (V)}⊇{\hat{A}}_{i+1}\left[H_{V}^{\left(n\right)}\setminus L_{V|Y_{\left(1\right)}}^{\left(n\right)}\right]$ (elements inside red curve at Block i+1 in Figure 3, Figure 4, Figure 5 and Figure 6). Therefore, Receiver 1 performs SC decoding to form ${\hat{A}}_{i+1}^{n}$ by using ${\hat{\Upsilon }}_{(1),i+1}^{\prime (V)}$, $\Phi_{(1),i+1}^{\left(V\right)}$ and the channel observations ${\tilde{Y}}_{(1),i+1}^{n}$.

#### 4.4.2. Legitimate Receiver 2

This receiver forms the estimates ${\hat{A}}_{1:L}^{n}$ by going backward, i.e., from ${\hat{A}}_{L}^{n}$ to ${\hat{A}}_{1}^{n}$, and this process is summarized in Algorithm 5.

**Algorithm 5** Decoding at legitimate Receiver 2**Require:**$\Upsilon_{\left(2\right)}^{\left(V\right)}$, $\Phi_{(2),1:L}^{\left(V\right)}$, $\kappa_{\Theta }^{\left(V\right)}$ and $\kappa_{\Gamma }^{\left(V\right)}$, and ${\tilde{Y}}_{(2),1:L}^{n}$.
 1:
${\hat{A}}_{L}^{n}\leftarrow \left(\Upsilon_{\left(2\right)}^{\left(V\right)},\Phi_{(2),L}^{\left(V\right)},{\tilde{Y}}_{(2),L}^{n}\right)$
 2:
${\hat{\Lambda }}_{1:L-1}^{\left(V\right)}\leftarrow {\hat{A}}_{L}\left[R_{\Lambda }^{\left(n\right)}\right]$
 3:**for**i=L to 2 **do** 4:    ${\hat{\bar{\Theta }}}_{i}^{\left(V\right)}\leftarrow {\hat{A}}_{i}\left[C_{1}^{\left(n\right)}\right]⊕\kappa_{\Theta }^{\left(V\right)}$ 5:    ${\hat{\bar{\Gamma }}}_{i}^{\left(V\right)}\leftarrow {\hat{A}}_{i}\left[C_{1,2}^{\left(n\right)}\right]⊕\kappa_{\Gamma }^{\left(V\right)}$ 6:    ${\hat{\Psi }}_{i-1}^{\left(V\right)}\leftarrow \left({\hat{A}}_{i}\left[R_{2}^{\left(n\right)}\right],{\hat{A}}_{i}\left[R_{1,2}^{\prime (n)}\right]⊕{\hat{\bar{\Theta }}}_{2,i+1}^{\left(V\right)}\right)$ 7:    ${\hat{\Gamma }}_{i-1}^{\left(V\right)}\leftarrow \left({\hat{A}}_{i}\left[R_{1,2}^{\left(n\right)}\right]⊕{\hat{\bar{\Gamma }}}_{1,i+1}^{\left(V\right)},{\hat{A}}_{i}\left[R_{2}^{\prime (n)}\right]\right)$ 8:    ${\hat{\Pi }}_{i-1}^{\left(V\right)}\leftarrow {\hat{A}}_{i}\left[R_{S}^{\left(n\right)}\right]$ 9:    $\Upsilon_{(2),i-1}^{\prime (V)}\leftarrow \left({\hat{\bar{\Theta }}}_{1,i}^{\left(V\right)},{\hat{\bar{\Gamma }}}_{2,i}^{\left(V\right)},{\hat{\Psi }}_{i-1}^{\left(V\right)},{\hat{\Gamma }}_{i-1}^{\left(V\right)},{\hat{\Pi }}_{i-1}^{\left(V\right)},{\hat{\Lambda }}_{i-1}^{\left(V\right)}\right)$10:    ${\hat{A}}_{i-1}^{n}\leftarrow \left(\Upsilon_{(2),i-1}^{\prime (V)},\Phi_{(2),i-1}^{\left(V\right)},{\tilde{Y}}_{(2),i-1}^{n}\right)$11:
**end for**



In all cases (among Case A to Case D), Receiver 2 constructs ${\hat{A}}_{L}^{n}$ as follows. Given $\Upsilon_{\left(2\right)}^{\left(V\right)}$ (all the elements inside blue curve at Block 4 in Figure 3, Figure 4, Figure 5 and Figure 6) and $\Phi_{(2),L}^{\left(V\right)}$, notice that Receiver 2 knows ${\tilde{A}}_{L}\left[{\left(L_{V|Y_{\left(2\right)}}^{\left(n\right)}\right)}^{C}\right]$. Hence, from $(\Upsilon_{\left(2\right)}^{\left(V\right)}$, $\Phi_{(2),L}^{\left(V\right)})$ and channel output observations ${\tilde{Y}}_{(2),L}^{n}$, Receiver 2 performs SC decoding to form ${\hat{A}}_{L}^{n}$. Since $\Lambda_{1}^{\left(V\right)}$ has been replicated in all blocks, from ${\hat{A}}_{L}^{n}$ it obtains ${\hat{\Lambda }}_{1:L-1}^{\left(V\right)}={\hat{A}}_{L}\left[R_{\Lambda }^{\left(n\right)}\right]$ (gray pentagons at all blocks).

For i∈[2,L], consider the construction of ${\hat{A}}_{i-1}^{n}$. First, since ${\hat{A}}_{i:L}^{n}$ have already been estimated, from ${\hat{A}}_{i}^{n}$ Receiver 2 obtains the sequence ${\hat{\Theta }}_{i}^{\left(V\right)}={\hat{A}}_{i}\left[C_{1}^{\left(n\right)}\right]$ (e.g., yellow squares at Block 3 in Figure 3, Figure 4, Figure 5 and Figure 6). Given ${\hat{\Theta }}_{i}^{\left(V\right)}$, it computes ${\hat{\bar{\Theta }}}_{i}^{\left(V\right)}={\hat{\Theta }}_{i}^{\left(V\right)}⊕\kappa_{\Theta }^{\left(V\right)}$ (yellow squares with a line through them). Furthermore, Receiver 2 obtains ${\hat{\Gamma }}_{i}^{\left(V\right)}={\hat{A}}_{i}\left[C_{1,2}^{\left(n\right)}\right]$ (yellow triangles at Block 3 in Figure 3, Figure 4, Figure 5 and Figure 6). Given this sequence, it computes ${\hat{\bar{\Gamma }}}_{i}^{\left(V\right)}={\hat{\Gamma }}_{i}^{\left(V\right)}⊕\kappa_{\Gamma }^{\left(V\right)}$ (yellow triangles with a line through them).

Furthermore, from ${\hat{A}}_{i}^{n}$, Receiver 2 obtains ${\hat{\Psi }}_{i-1}^{\left(V\right)}$ as follows. At block *L*, in all cases it gets ${\hat{\Psi }}_{L-1}^{\left(V\right)}={\hat{A}}_{i}\left[R_{2}^{\left(n\right)}\cup R_{1,2}^{\prime (n)}\right]$ directly (all yellow circles at Block *L* in Figure 3, Figure 4, Figure 5 and Figure 6). At Block i∈[2,L−1], we distinguish two situations:

- In Case D, Receiver 2 obtains ${\hat{\Psi }}_{1,i-1}^{\left(V\right)}={\hat{A}}_{i}\left[R_{2}^{\left(n\right)}\right]$ (e.g., red circles at Block 3 in Figure 6) and ${\hat{\Psi }}_{2,i-1}^{\left(V\right)}⊕{\hat{\bar{\Theta }}}_{2,i+1}^{\left(V\right)}={\hat{A}}_{i}\left[R_{1,2}^{\prime (n)}\right]$ (cyan squares with a line through them overlapped by red circles). Since ${\hat{\bar{\Theta }}}_{2,i+1}^{\left(V\right)}$ (cyan squares with a line through them) has already been estimated, it obtains ${\hat{\Psi }}_{2,i-1}^{\left(V\right)}={\hat{A}}_{i}\left[R_{1,2}^{\prime (n)}\right]⊕{\hat{\bar{\Theta }}}_{2,i+1}^{\left(V\right)}$ (red circles).
- Otherwise, in other cases, Receiver 2 obtains directly ${\hat{\Psi }}_{i-1}^{\left(V\right)}={\hat{A}}_{i}\left[R_{2}^{\left(n\right)}\right]$ (e.g., red circles at Block 3 in Figure 3, Figure 4 and Figure 5).

From ${\hat{A}}_{i}^{n}$, Receiver 2 also obtains ${\hat{\Gamma }}_{i-1}^{\left(V\right)}$ as follows. At block *L*, in all cases it gets ${\hat{\bar{\Gamma }}}_{L-1}^{\left(V\right)}={\hat{A}}_{L}\left[R_{1,2}^{\left(n\right)}\cup R_{2}^{\prime (n)}\right]$ (e.g., all yellow triangles at Block *L* in Figure 3, Figure 4, Figure 5 and Figure 6). At Block i∈[2,L−1], in all cases Receiver 2 obtains ${\hat{\Gamma }}_{1,i-1}^{\left(V\right)}⊕{\hat{\bar{\Gamma }}}_{1,i+1}^{\left(V\right)}={\hat{A}}_{i}\left[R_{1,2}^{\left(n\right)}\right]$ (e.g., red and cyan diamonds with a line through them at Block 3). Since ${\hat{\bar{\Gamma }}}_{1,i+1}^{\left(V\right)}$ (cyan triangles with a line through them) has already been estimated, Receiver 2 obtains ${\hat{\Gamma }}_{1,i-1}^{\left(V\right)}={\hat{A}}_{i}\left[R_{1,2}^{\left(n\right)}\right]⊕{\hat{\bar{\Gamma }}}_{1,i+1}^{\left(V\right)}$ (red triangles). Furthermore, only in Case B, Receiver 2 obtains the sequence ${\hat{\Gamma }}_{2,i-1}^{\left(V\right)}={\hat{A}}_{i}\left[R_{2}^{\prime (n)}\right]$ (remaining red triangles at Block 3 in Figure 4).

Lastly, only in Case A and Case B, Receiver 2 obtains the sequence ${\hat{\Pi }}_{i-1}^{\left(V\right)}={\hat{A}}_{i}\left[R_{S}^{\left(n\right)}\right]$ (e.g., purple crosses at Block 3 in Figure 3 and Figure 4).

Finally, define the sequence $\Upsilon_{(2),i-1}^{\prime (V)}≜\left[{\hat{\bar{\Theta }}}_{1,i}^{\left(V\right)},{\hat{\bar{\Gamma }}}_{2,i}^{\left(V\right)},{\hat{\Psi }}_{i-1}^{\left(V\right)},{\hat{\Gamma }}_{i-1}^{\left(V\right)},{\hat{\Pi }}_{i-1}^{\left(V\right)},{\hat{\Lambda }}_{i-1}^{\left(V\right)}\right]$. Notice that $\Upsilon_{(2),i-1}^{\prime (V)}⊇{\hat{A}}_{i-1}\left[H_{V}^{\left(n\right)}\setminus L_{V|Y_{\left(2\right)}}^{\left(n\right)}\right]$ (elements inside blue curve at Block i−1 in Figure 3, Figure 4, Figure 5 and Figure 6). Thus, Receiver 2 performs SC decoding to form ${\hat{A}}_{i-1}^{n}$ by using $\Upsilon_{(2),i-1}^{\prime (V)}$, $\Phi_{(2),i-1}^{\left(V\right)}$ and ${\tilde{Y}}_{(2),i-1}^{n}$.

## 5. Performance of the Polar Coding Scheme

The analysis of the polar coding scheme of Section 4 leads to the following theorem.

**Theorem** **1.** *Let $\left(X,p_{Y_{\left(1\right)}Y_{\left(2\right)}Z|X},Y_{\left(1\right)}\times Y_{\left(2\right)}\times Z\right)$ be an arbitrary WTBC, such that X∈{0,1}. The polar coding scheme described in Section 4 achieves the corner point in Equation *(4)* of the region $R_{\mathrm{CI}-\mathrm{WTBC}}$ defined in Proposition 1.*

The proof of Theorem 1 follows in four steps and is provided in the following subsections. In Section 5.1 we show that the polar coding scheme approaches the rate tuple in (4). In Section 5.2 we prove that the joint distribution of $\left({\tilde{V}}_{i}^{n},{\tilde{X}}_{i}^{n},{\tilde{Y}}_{(1),i}^{n},{\tilde{Y}}_{(2),i}^{n},{\tilde{Z}}_{i}^{n}\right)$, for all i∈[1,L], is asymptotically indistinguishable of the one of the original DMS that is used for the polar code construction. Finally, in Section 5.3 and Section 5.4 we show that the polar coding scheme satisfies the reliability and the secrecy conditions (1) and (2) respectively.

### 5.1. Transmission Rates

We prove that the polar coding scheme described in Section 4 approaches the rate tuple in Equation (4). Furthermore, we show that the overall length of the secret keys $\kappa_{\Theta }^{\left(V\right)}$, $\kappa_{\Gamma }^{\left(V\right)}$, $\kappa_{\Upsilon \Phi_{\left(1\right)}}^{\left(V\right)}$ and $\kappa_{\Upsilon \Phi_{\left(2\right)}}^{\left(V\right)}$, and the additional randomness used in the encoding (besides the randomization sequences) are asymptotically negligible in terms of rate.

#### 5.1.1. Private Message Rate

For i∈[1,L], we have $W_{i}={\tilde{A}}_{i}\left[C^{\left(n\right)}\right]$. According to the definition of $C^{\left(n\right)}$ in (11), and since $H_{V|Z}^{\left(n\right)}\subseteq H_{V}^{\left(n\right)}$, the rate of $W_{1:L}$ is

$$\begin{array}{c} \frac{1}{nL}\sum_{i=1}^{L}\left|W_{i}\right|=\frac{1}{n}|H_{V}^{\left(n\right)}\cap {\left(H_{V|Z}^{\left(n\right)}\right)}^{C}|=\frac{1}{n}|H_{V}^{\left(n\right)}|-\frac{1}{n}|H_{V|Z}^{\left(n\right)}|\overset{n\to \infty }{\to }H\left(V\right)-H\left(V|Z\right) \end{array}$$

where the limit holds by Lemma 4 of [10]. Therefore, the private message rate achieved by the polar coding scheme is $R_{W}=I\left(V;Z\right)$, as in (4).

#### 5.1.2. Confidential Message Rate

From Section 4.2, in all cases we have $S_{1}={\tilde{A}}_{1}\left[I^{\left(n\right)}\cup G_{1}^{\left(n\right)}\cup G_{1,2}^{\left(n\right)}\right]$; for i∈[2,L−1], we have $S_{i}={\tilde{A}}_{i}\left[I^{\left(n\right)}\right]$; and $S_{L}={\tilde{A}}_{L}\left[I^{\left(n\right)}\cup G_{2}^{\left(n\right)}\right]$. Thus, we have

$$\begin{array}{cc} \; & \frac{1}{nL}\sum_{i=1}^{L}\left|S_{i}\right|\\ \; & \;=\frac{\left(L-2\right)}{nL}|I^{\left(n\right)}|+\frac{1}{nL}\left(|I^{\left(n\right)}\cup G_{1}^{\left(n\right)}\cup G_{1,2}^{\left(n\right)}|+|I^{\left(n\right)}\cup G_{2}^{\left(n\right)}|\right)\\ \; & \;=\frac{1}{n}|I^{\left(n\right)}|+\frac{1}{nL}\left(|G_{1}^{\left(n\right)}|+|G_{2}^{\left(n\right)}|+|G_{1,2}^{\left(n\right)}|\right)\\ \; & \;=\frac{1}{n}|I^{\left(n\right)}|+\frac{1}{nL}|G^{\left(n\right)}\setminus G_{0}^{\left(n\right)}|\\ \; & \;\overset{\left(a\right)}{=}\frac{1}{n}\left(|G_{0}^{\left(n\right)}|+|G_{2}^{\left(n\right)}|-|R_{1,2}^{\left(n\right)}|-|R_{1,2}^{\prime (n)}|-|R_{1}^{\left(n\right)}|-|R_{1}^{\prime (n)}|\right)+\frac{1}{nL}|G^{\left(n\right)}\setminus G_{0}^{\left(n\right)}|\\ \; & \;\overset{\left(b\right)}{=}\frac{1}{n}\left(|G_{0}^{\left(n\right)}|+|G_{2}^{\left(n\right)}|-|C_{1}^{\left(n\right)}|-|C_{1,2}^{\left(n\right)}|\right)+\frac{\left|G^{\left(n\right)}\setminus G_{0}^{\left(n\right)}\right|}{nL}\\ \; & \;\overset{\left(c\right)}{\geq }\frac{1}{n}\left(|H_{V|Z}^{\left(n\right)}\cap L_{V|Y_{\left(1\right)}}^{\left(n\right)}|-|{\left(H_{V|Z}^{\left(n\right)}\right)}^{C}\cap {\left(L_{V|Y_{\left(1\right)}}^{\left(n\right)}\right)}^{C}|\right)+\frac{1}{nL}|H_{V|Z}^{\left(n\right)}\cap {\left(L_{V|Y_{\left(1\right)}}^{\left(n\right)}\cap L_{V|Y_{\left(2\right)}}^{\left(n\right)}\right)}^{C}|\\ \; & \;\overset{\left(d\right)}{\geq }\frac{1}{n}\left(|H_{V|Z}^{\left(n\right)}\cap L_{V|Y_{\left(1\right)}}^{\left(n\right)}|-|{\left(H_{V|Z}^{\left(n\right)}\right)}^{C}\cap {\left(L_{V|Y_{\left(1\right)}}^{\left(n\right)}\right)}^{C}|\right)+\frac{1}{nL}|H_{V|Z}^{\left(n\right)}|-\frac{1}{nL}|{\left(L_{V|Y_{\left(1\right)}}^{\left(n\right)}\right)}^{C}|\\ \; & \;=\frac{1}{n}|H_{V|Z}^{\left(n\right)}|-\frac{1}{n}|{\left(L_{V|Y_{\left(1\right)}}^{\left(n\right)}\right)}^{C}|+\frac{1}{nL}|H_{V|Z}^{\left(n\right)}|-\frac{1}{nL}|{\left(L_{V|Y_{\left(1\right)}}^{\left(n\right)}\right)}^{C}|\\ \; & \;\overset{n\to \infty }{\to }H\left(V|Z\right)-H\left(V|Y_{\left(1\right)}\right)+\frac{1}{L}\left(H\left(V|Z\right)-H\left(V|Y_{\left(1\right)}\right)\right)\\ \; & \;\overset{L\to \infty }{\to }H\left(V|Z\right)-H\left(V|Y_{\left(1\right)}\right) \end{array}$$

where (a) holds by the definition of $I^{\left(n\right)}$ in (24); (b) holds because, in all cases, we have $\left|R_{1,2}^{\left(n\right)}|+|R_{1}^{\prime (n)}|=|C_{1,2}^{\left(n\right)}\right|$ and $\left|R_{1}^{\left(n\right)}|+|R_{1,2}^{\prime (n)}|=|C_{1}^{\left(n\right)}\right|$; (c) follows from the partition of $H_{V}^{\left(n\right)}$ defined in (12)–(19); (d) follows from applying elementary set operations and because, by assumption, $H\left(V|Y_{\left(1\right)}\right)\geq H\left(V|Y_{\left(2\right)}\right)$, which means that $\left|{\left(L_{V|Y_{\left(1\right)}}^{\left(n\right)}\right)}^{C}|\geq |{\left(L_{V|Y_{\left(2\right)}}^{\left(n\right)}\right)}^{C}\right|$ (by Lemma 4 of [10]); and the limit when *n* goes to infinity holds also by Lemma 4 of [10]. Hence, the polar coding scheme operates as close to the rate $R_{S}$ in (4) as desired by choosing a sufficiently large *L*.

#### 5.1.3. Randomization Sequence Rate

For i∈[1,L], we have $R_{i}={\tilde{T}}_{i}\left[H_{X|V}^{\left(n\right)}\cap {\left(H_{X|VZ}^{\left(n\right)}\right)}^{C}\right]$. Since $H_{X|VZ}^{\left(n\right)}⊇H_{X|V}^{\left(n\right)}$, we have

$$\begin{array}{cc} \frac{1}{nL}\sum_{i=1}^{L}\left|R_{i}\right| & =\frac{1}{n}|H_{X|V}^{\left(n\right)}\cap {\left(H_{X|VZ}^{\left(n\right)}\right)}^{C}|=\frac{1}{n}|H_{X|V}^{\left(n\right)}|-\frac{1}{n}|H_{X|VZ}^{\left(n\right)}|\overset{n\to \infty }{\to }H\left(X|Z\right)-H\left(X|VZ\right) \end{array}$$

where the limit holds by Lemma 4 of [10]. Thus, the randomization sequence rate used by the polar coding scheme is $R_{R}=I\left(X;Z|V\right)$ as in (4).

#### 5.1.4. Private-Shared Sequence Rate

Transmitter and legitimate Receiver k∈[1,2] must privately share the keys $\kappa_{\Theta }^{\left(V\right)}$, $\kappa_{\Gamma }^{\left(V\right)}$ and $\kappa_{\Upsilon \Phi_{\left(k\right)}}^{\left(V\right)}$. Hence, the overall rate is

$$\begin{array}{cc} \; & \frac{1}{nL}\left(|\kappa_{\Theta }^{\left(V\right)}|+|\kappa_{\Gamma }^{\left(V\right)}|+\sum_{k=1}^{2}|\kappa_{\Upsilon \Phi_{\left(k\right)}}^{\left(V\right)}|\right)\\ \; & \;=\frac{1}{nL}\left(|C_{1}^{\left(n\right)}|+|C_{1,2}^{\left(n\right)}|\right)+\frac{1}{nL}\sum_{k=1}^{2}\left(L|{\left(H_{V}^{\left(n\right)}\right)}^{C}\cap {\left(L_{V|Y_{\left(k\right)}}^{\left(n\right)}\right)}^{C}|+|H_{V}^{\left(n\right)}\cap {\left(L_{V|Y_{\left(k\right)}}^{\left(n\right)}\right)}^{C}|\right)\\ \; & \;\overset{\left(a\right)}{=}\frac{1}{nL}\left(|H_{V}^{\left(n\right)}\cap {\left(H_{V|Z}^{\left(n\right)}\right)}^{C}\cap {\left(L_{V|Y_{\left(1\right)}}^{\left(n\right)}\right)}^{C}|\right)\\ \; & \;+\frac{1}{nL}\sum_{k=1}^{2}\left(L|{\left(H_{V}^{\left(n\right)}\right)}^{C}\cap {\left(L_{V|Y_{\left(k\right)}}^{\left(n\right)}\right)}^{C}|+|H_{V}^{\left(n\right)}\cap {\left(L_{V|Y_{\left(k\right)}}^{\left(n\right)}\right)}^{C}|\right)\\ \; & \;\overset{\left(b\right)}{\leq }\frac{1}{nL}|{\left(L_{V|Y_{\left(1\right)}}^{\left(n\right)}\right)}^{C}|+\frac{1}{nL}\sum_{k=1}^{2}\left(L|{\left(H_{V|Y_{\left(k\right)}}^{\left(n\right)}\right)}^{C}\cap {\left(L_{V|Y_{\left(k\right)}}^{\left(n\right)}\right)}^{C}|+|{\left(L_{V|Y_{\left(k\right)}}^{\left(n\right)}\right)}^{C}|\right)\\ \; & \;\overset{n\to \infty }{\to }\frac{1}{L}\left(2H\left(V|Y_{\left(1\right)}\right)+H\left(V|Y_{\left(2\right)}\right)\right)\\ \; & \;\overset{L\to \infty }{\to }0, \end{array}$$

where (a) follows from the definition of $C_{1}^{\left(n\right)}$ and $C_{1,2}^{\left(n\right)}$ in (17) and (19), respectively; (b) follows from standard set properties and because ${\left(H_{V|Z}^{\left(n\right)}\right)}^{C}\subseteq {\left(H_{V|Y_{\left(k\right)}}^{\left(n\right)}\right)}^{C}$ for any k∈[1,2]; and the limit when *n* goes to infinity holds by Lemma 4 of [10].

#### 5.1.5. Rate of the Additional Randomness

Besides the randomization sequences $R_{1:L}$, the encoder uses the random sequence $\Lambda_{0}^{\left(X\right)}$, with size $\left|H_{X|V}^{\left(n\right)}\right|$, for the polar-based channel prefixing. Moreover, for i∈[1,L], the encoder randomly draws those elements ${\tilde{A}}_{i}\left(j\right)$ such that $j\in {\left(H_{V}^{\left(n\right)}\right)}^{C}\setminus L_{V}^{\left(n\right)}$, and those elements ${\tilde{T}}_{i}\left(j\right)$ such that $j\in {\left(H_{X|V}^{\left(n\right)}\right)}^{C}\setminus L_{X|V}^{\left(n\right)}$. Nevertheless, we have

$$\begin{array}{cc} \; & \frac{1}{nL}\left(|H_{X|V}^{\left(n\right)}|+L|{\left(H_{V}^{\left(n\right)}\right)}^{C}\setminus L_{V}^{\left(n\right)}|+L|{\left(H_{X|V}^{\left(n\right)}\right)}^{C}\setminus L_{X|V}^{\left(n\right)}|\right)\\ \; & \;\overset{n\to \infty }{\to }\frac{1}{L}H\left(X|V\right)\\ \; & \;\overset{L\to \infty }{\to }0, \end{array}$$

where the limit when *n* approaches to infinity follows from applying Lemma 4 of [10].

### 5.2. Distribution of the DMS after the Polar Encoding

For i∈[1,L], let ${\tilde{q}}_{A_{i}^{n}T_{i}^{n}}$ denote the distribution of $\left({\tilde{A}}_{i}^{n},{\tilde{T}}_{i}^{n}\right)$ after the encoding. The following lemma proves that ${\tilde{q}}_{A_{i}^{n}T_{i}^{n}}$ and the marginal distribution $p_{A^{n}T^{n}}$ of the original DMS are nearly statistically indistinguishable for sufficiently large *n* and, consequently, so are ${\tilde{q}}_{V_{i}^{n}X_{i}^{n}Y_{(1),i}^{n}Y_{(2),i}^{n}Z_{i}^{n}}$ and $p_{V^{n}X^{n}Y_{\left(1\right)}^{n}Y_{\left(2\right)}^{n}Z^{n}}$. This result is crucial for the reliability and secrecy performance of the polar coding scheme.

**Lemma** **1.** *For any i∈[1,L], we obtain*

$$\begin{array}{cc} V({\tilde{q}}_{A_{i}^{n}T_{i}^{n}},p_{A^{n}T^{n}}) & \leq \delta_{n}^{\left(*\right)},\\ V({\tilde{q}}_{V_{i}^{n}X_{i}^{n}Y_{(1),i}^{n}Y_{(2),i}^{n}Z_{i}^{n}},p_{V^{n}X^{n}Y_{\left(1\right)}^{n}Y_{\left(2\right)}^{n}Z^{n}}) & \leq \delta_{n}^{\left(*\right)}, \end{array}$$

where $\delta_{n}^{\left(*\right)}≜2n\sqrt{4\sqrt{n\delta_{n}\ln2}\left(2n-\log\left(2\sqrt{n\delta_{n}\ln2}\right)\right)+\delta_{n}}+2\sqrt{n\delta_{n}\ln2}$.

**Proof.**  Omitted because it follows similar reasoning as in Lemma 3 of [11]. □

### 5.3. Reliability Analysis

In this section we prove that both legitimate receivers can reliably reconstruct the private and the confidential messages $\left(W_{1:L},S_{1:L}\right)$ with arbitrary small error probability.

For i∈[1,L] and k∈[1,2], let ${\tilde{q}}_{V_{i}^{n}Y_{(k),i}^{n}}$ and $p_{V^{n}Y_{\left(k\right)}^{n}}$ be marginals of ${\tilde{q}}_{V_{i}^{n}X_{i}^{n}Y_{(1),i}^{n}Y_{(2),i}^{n}Z_{i}^{n}}$ and $p_{V^{n}X^{n}Y_{\left(1\right)}^{n}Y_{\left(2\right)}^{n}Z^{n}}$ respectively, and define an optimal coupling Proposition 4.7 of [25] between ${\tilde{q}}_{V_{i}^{n}Y_{(k),i}^{n}}$ and $p_{V^{n}Y_{\left(k\right)}^{n}}$ such that $P\left[E_{V_{i}^{n}Y_{(k),i}^{n}}\right]=V\left({\tilde{q}}_{V_{i}^{n}Y_{(k),i}^{n}},p_{V^{n}Y_{\left(k\right)}^{n}}\right)$, where $E_{V_{i}^{n}Y_{(k),i}^{n}}≜\left\{\left({\tilde{V}}_{i}^{n},{\tilde{Y}}_{(k),i}^{n}\right)\neq \left(V^{n},Y_{\left(k\right)}^{n}\right)\right\}$. Additionally, define the error event

$$\begin{array}{c} E_{(k),i}≜\left\{{\hat{A}}_{i}\left[{\left(L_{V|Y_{\left(k\right)}}^{\left(n\right)}\right)}^{C}\right]\neq {\tilde{A}}_{i}\left[{\left(L_{V|Y_{\left(k\right)}}^{\left(n\right)}\right)}^{C}\right]\right\}. \end{array}$$

Recall that $\left(\Upsilon_{\left(k\right)}^{\left(V\right)},\Phi_{(k),1:L}^{\left(V\right)}\right)$ is available to Receiver k∈[1,2]. Thus, $P\left[E_{(1),1}\right]=P\left[E_{(2),L}\right]=0$ because given $\Upsilon_{\left(1\right)}^{\left(V\right)}$ and $\Phi_{(1),1}^{\left(V\right)}$ legitimate Receiver 1 knows ${\tilde{A}}_{1}\left[{\left(L_{V|Y_{\left(1\right)}}^{\left(n\right)}\right)}^{C}\right]$, and given $\Upsilon_{\left(2\right)}^{\left(V\right)}$ and $\Phi_{(2),L}^{\left(V\right)}$ legitimate Receiver 2 knows ${\tilde{A}}_{L}\left[{\left(L_{V|Y_{\left(2\right)}}^{\left(n\right)}\right)}^{C}\right]$. Moreover, due to the chaining structure, in Section 4.4 we have seen that ${\tilde{A}}_{i}\left[H_{V}^{\left(n\right)}\cap {\left(L_{V|Y_{\left(1\right)}}^{\left(n\right)}\right)}^{C}\right]$ is repeated in ${\tilde{A}}_{i-1}^{n}$ for i∈[2,L]. Therefore, at legitimate Receiver 1, for i∈[2,L] we have

(35) $$\begin{array}{c} P\left[E_{(1),i}\right]\leq P\left[{\hat{A}}_{i-1}^{n}\neq {\tilde{A}}_{i-1}^{n}\right]. \end{array}$$

Similarly, due to the chaining construction, we have seen that ${\tilde{A}}_{i}\left[H_{V}^{\left(n\right)}\cap {\left(L_{V|Y_{\left(2\right)}}^{\left(n\right)}\right)}^{C}\right]$ is repeated in ${\tilde{A}}_{i+1}^{n}$ for i∈[1,L−1]. Thus, at legitimate Receiver 2, for i∈[1,L−1] we obtain

(36) $$\begin{array}{c} P\left[E_{(2),i}\right]\leq P\left[{\hat{A}}_{i+1}^{n}\neq {\tilde{A}}_{i+1}^{n}\right]. \end{array}$$

Hence, the probability of incorrectly decoding $\left(W_{i},S_{i}\right)$ at the Receiver k∈[1,2] is

$$\begin{array}{cc} P\left[\left(W_{i},S_{i}\right)\neq \left({\hat{W}}_{i},{\hat{S}}_{i}\right)\right] & \leq P\left[{\hat{A}}_{i}^{n}\neq {\tilde{A}}_{i}^{n}\right]\\ \; & =P\left[{\hat{A}}_{i}^{n}\neq {\tilde{A}}_{i}^{n}|E_{V_{i}^{n}Y_{(k),i}^{n}}^{C}\cap E_{(k),i}^{C}\right]P\left[E_{V_{i}^{n}Y_{(k),i}^{n}}^{C}\cap E_{(k),i}^{C}\right]\\ \; & \;+P\left[{\hat{A}}_{i}^{n}\neq {\tilde{A}}_{i}^{n}|E_{V_{i}^{n}Y_{(k),i}^{n}}\cup E_{(k),i}\right]P\left[E_{V_{i}^{n}Y_{(k),i}^{n}}\cup E_{(k),i}\right]\\ \; & \leq P\left[{\hat{A}}_{i}^{n}\neq {\tilde{A}}_{i}^{n}|E_{V_{i}^{n}Y_{(k),i}^{n}}^{C}\cap E_{(k),i}^{C}\right]+P\left[E_{V_{i}^{n}Y_{(k),i}^{n}}\right]+P\left[E_{(k),i}\right]\\ \; & \overset{\left(a\right)}{\leq }n\delta_{n}+P\left[E_{V_{i}^{n}Y_{(k),i}^{n}}\right]+P\left[E_{(k),i}\right]\\ \; & \overset{\left(b\right)}{\leq }n\delta_{n}+\delta_{n}^{\left(*\right)}+P\left[E_{(k),i}\right]\\ \; & \overset{\left(c\right)}{\leq }i\left(n\delta_{n}+\delta_{n}^{\left(*\right)}\right), \end{array}$$

where (a) holds by Th. 2 of [19]; (b) follows from the optimal coupling and Lemma 1; and (c) holds by induction and Equations (35) and (36). Therefore, by the union bound we obtain

$$\begin{array}{c} P\left[\left(W_{1:L},S_{1:L}\right)\neq \left({\hat{W}}_{1:L},{\hat{S}}_{1:L}\right)\right]\leq \sum_{i=1}^{L}P\left[{\tilde{A}}_{i}^{n}\neq {\hat{A}}_{i}^{n}\right]\leq \frac{L(L+1)}{2}\left(n\delta_{n}+2\delta_{n}^{\left(*\right)}\right), \end{array}$$

and for sufficiently large *n* the polar coding scheme satisfies the reliability condition in (1).

### 5.4. Secrecy Analysis

Since encoding in Section 4 takes place over *L* blocks of size *n*, we need to prove that

$$\begin{array}{c} \lim_{n\to \infty }I\left(S_{1:L},{\tilde{Z}}_{1:L}^{n}\right)=0. \end{array}$$

**Figure 7:**
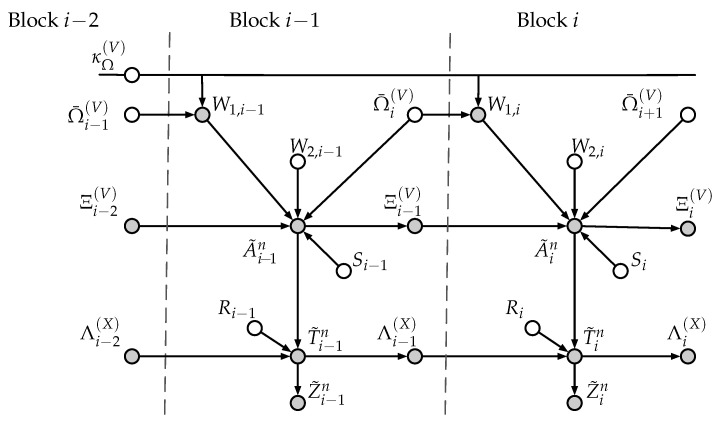
Graphical representation (Bayesian graph) of the dependencies between random variables involved in the polar coding scheme. Independent random variables are indicated by white nodes, whereas those that are dependent are indicated by gray nodes.

For clarity and with slight abuse of notation, for any Block i∈[1,L] let

$$\begin{array}{c} \Xi_{i}^{\left(V\right)}≜\left[\Pi_{i}^{\left(V\right)},\Lambda_{i}^{\left(V\right)},\Psi_{i}^{\left(V\right)},\Gamma_{i}^{\left(V\right)}\right], \end{array}$$

which denotes the entire sequence depending on ${\tilde{A}}_{i}^{n}$ that is repeated at Block i+1. Furthermore, let

$$\begin{array}{c} {\bar{\Omega }}_{i}^{\left(V\right)}≜\left[{\bar{\Theta }}_{i}^{\left(V\right)},{\bar{\Gamma }}_{i}^{\left(V\right)}\right], \end{array}$$

which represents the sequence depending on ${\tilde{A}}_{i}^{n}$ that is repeated at Block i−1. Furthermore, we define $\kappa_{\Omega }^{\left(V\right)}≜\left[\kappa_{\Theta }^{\left(V\right)},\kappa_{\Gamma }^{\left(V\right)}\right]$. Then, a Bayesian graph describing the dependencies between all the variables involved in the polar coding scheme of Section 4 is given in Figure 7.

Despite $\Gamma_{i}^{\left(V\right)}\subseteq \Xi_{i}^{\left(V\right)}$ and ${\bar{\Gamma }}_{i}^{\left(V\right)}=\Gamma_{i}^{\left(V\right)}⊕\kappa_{\Gamma }^{\left(V\right)}\subseteq {\bar{\Omega }}_{i}^{\left(V\right)}$, we represent $\Xi_{i}^{\left(V\right)}$ and ${\bar{\Omega }}_{i}^{\left(V\right)}$ as two separate nodes in the Bayesian graph because, by *crypto lemma* [26], $\Gamma_{i}^{\left(V\right)}$ and ${\bar{\Gamma }}_{i}^{\left(V\right)}$ are statistically independent. Furthermore, for convenience, we have considered that dependencies only take place forward (from Block *i* to Block i+1), which is possible by reformulating the encoding as follows. According to Section 4.1, for any i∈[1,L] we have ${\tilde{A}}_{i}\left[C^{\left(n\right)}\right]=W_{i}$. Consequently, we can write $W_{i}≜\left[W_{1,i},W_{2,i}\right]$, where $W_{1,i}≜{\tilde{A}}_{i}\left[C_{1}^{\left(n\right)}\cup C_{1,2}^{\left(n\right)}\right]$ and $W_{2,i}≜{\tilde{A}}_{i}\left[C_{2}^{\left(n\right)}\cup C_{0}^{\left(n\right)}\right]$. Since ${\bar{\Theta }}_{i}^{\left(V\right)}={\tilde{A}}_{i}\left[C_{1}^{\left(n\right)}\right]⊕\kappa_{\Theta }^{\left(V\right)}$ and ${\bar{\Gamma }}_{i}^{\left(V\right)}={\tilde{A}}_{i}\left[C_{1,2}^{\left(n\right)}\right]⊕\kappa_{\Gamma }^{\left(V\right)}$, we regard ${\bar{\Omega }}_{i}^{\left(V\right)}$ as an independent random sequence generated at Block i−1 that is stored properly into some part of ${\tilde{A}}_{i-1}\left[G^{\left(n\right)}\right]$. Then, we consider that the encoder obtains $W_{1,i}≜{\bar{\Omega }}_{i}^{\left(V\right)}⊕\kappa_{\Omega }^{\left(V\right)}$, which is stored into ${\tilde{A}}_{i}\left[C_{1}^{\left(n\right)}\cup C_{1,2}^{\left(n\right)}\right]$ at Block *i*. On the other hand, the remaining part $W_{2,i}$ is independently generated at Block *i*. Recall that the *secret-key*$\kappa_{\Omega }^{\left(V\right)}$ is reused in all blocks.

The following lemma shows that strong secrecy holds for any Block i∈[1,L].

**Lemma** **2.** *For any i∈[1,L] and sufficiently large n,*

$$\begin{array}{c} I\left(S_{i}\Xi_{i-1}^{\left(V\right)}\Lambda_{i-1}^{\left(X\right)};{\tilde{Z}}_{i}^{n}\right)\leq \delta_{n}^{\left(S\right)}, \end{array}$$

where $\delta_{n}^{\left(S\right)}≜2n\delta_{n}+2\delta_{n}^{\left(*\right)}\left(2n-\log\delta_{n}^{\left(*\right)}\right)$ and $\delta_{n}^{\left(*\right)}$ defined as in Lemma 1.

**Proof.**  For *n* sufficiently large, we have

$$\begin{array}{cc} \; & I\left(S_{i}\Xi_{i-1}^{\left(V\right)}\Lambda_{i-1}^{\left(X\right)};{\tilde{Z}}_{i}^{n}\right)\\ \; & \;\overset{\left(a\right)}{=}I\left({\tilde{A}}_{i}\left[H_{V|Z}^{\left(n\right)}\right]{\tilde{T}}_{i}\left[H_{X|VZ}^{\left(n\right)}\right];{\tilde{Z}}_{i}^{n}\right)\\ \; & \;\overset{\left(b\right)}{=}|H_{V|Z}^{\left(n\right)}|+|H_{X|VZ}^{\left(n\right)}|-H\left({\tilde{A}}_{i}\left[H_{V|Z}^{\left(n\right)}\right]{\tilde{T}}_{i}\left[H_{X|VZ}^{\left(n\right)}\right]|{\tilde{Z}}_{i}^{n}\right)\\ \; & \;\overset{\left(c\right)}{\leq }|H_{V|Z}^{\left(n\right)}|+|H_{X|VZ}^{\left(n\right)}|-H\left(A\left[H_{V|Z}^{\left(n\right)}\right]T\left[H_{X|VZ}^{\left(n\right)}\right]|Z_{i}^{n}\right)+4n\delta_{n}^{\left(*\right)}-2\delta_{n}^{\left(*\right)}\log\delta_{n}^{\left(*\right)}\\ \; & \;\overset{\left(d\right)}{\leq }2n\delta_{n}+4n\delta_{n}^{\left(*\right)}-2\delta_{n}^{\left(*\right)}\log\delta_{n}^{\left(*\right)} \end{array}$$

where (a) holds by the encoding described in Section 4; (b) holds by the uniformity of ${\tilde{A}}_{i}\left[H_{V|Z}^{\left(n\right)}\right]$ and ${\tilde{A}}_{i}\left[H_{X|VZ}^{\left(n\right)}\right]$; (c) holds because, for *n* large enough, we obtain

$$\begin{array}{cc} \; & \left|H\left({\tilde{A}}_{i}\left[H_{V|Z}^{\left(n\right)}\right]{\tilde{T}}_{i}\left[H_{X|VZ}^{\left(n\right)}\right]|{\tilde{Z}}_{i}^{n}\right)-H\left(A_{i}\left[H_{V|Z}^{\left(n\right)}\right]T_{i}\left[H_{X|VZ}^{\left(n\right)}\right]|Z_{i}^{n}\right)\right|\\ \; & \;\leq |H\left({\tilde{Z}}_{m}^{n}\right)-H\left(Z_{m}^{n}\right)|+|H\left({\tilde{A}}_{i}\left[H_{V|Z}^{\left(n\right)}\right]{\tilde{T}}_{i}\left[H_{X|VZ}^{\left(n\right)}\right]{\tilde{Z}}_{i}^{n}\right)-H\left(A_{i}\left[H_{V|Z}^{\left(n\right)}\right]T_{i}\left[H_{X|VZ}^{\left(n\right)}\right]Z_{i}^{n}\right)|\\ \; & \;\leq V\left({\tilde{q}}_{Z_{m}^{n}},p_{Z_{m}^{n}}\right)\log\frac{2^{n}}{V({\tilde{q}}_{Z_{m}^{n}},p_{Z_{m}^{n}})}\\ \; & \;+V\left({\tilde{q}}_{A_{i}[H_{V|Z}^{\left(n\right)}]T_{i}\left[H_{X|VZ}^{\left(n\right)}\right]Z^{n}},p_{A_{i}\left[H_{V|Z}^{\left(n\right)}\right]T_{i}\left[H_{X|VZ}^{\left(n\right)}\right]Z^{n}}\right)\log\frac{2^{\left(n+|H_{V|Z}^{\left(n\right)}|+|H_{X|VZ}^{\left(n\right)}|\right)}}{V\left({\tilde{q}}_{A_{i}\left[H_{V|Z}^{\left(n\right)}\right]T_{i}\left[H_{X|VZ}^{\left(n\right)}\right]Z^{n}},p_{A_{i}\left[H_{V|Z}^{\left(n\right)}\right]T_{i}\left[H_{X|VZ}^{\left(n\right)}\right]Z^{n}}\right)}\\ \; & \;\overset{\left(b\right)}{\leq }4n\delta_{\mathrm{ld}-\mathrm{nls}}^{\left(n\right)}-2\delta_{\mathrm{ld}-\mathrm{nls}}^{\left(n\right)}\log\delta_{\mathrm{ld}-\mathrm{nls}}^{\left(n\right)}, \end{array}$$

where we have used the chain rule of entropy and the triangle inequality, ([27], Lemma 30), the fact that the function x→xlogx is decreasing for x>0 small enough and Lemma 1; and, lastly, (d) holds because

$$\begin{array}{cc} \; & H\left(A\left[H_{V|Z}^{\left(n\right)}\right]T\left[H_{X|VZ}^{\left(n\right)}\right]|Z^{n}\right)\\ \; & \;\geq H\left(A\left[H_{V|Z}^{\left(n\right)}\right]|Z^{n}\right)+H\left(T\left[H_{X|VZ}^{\left(n\right)}\right]|A^{n}Z^{n}\right)\\ \; & \;\geq \sum_{j\in H_{V|Z}^{\left(n\right)}}H\left(A\left(j\right)|A^{1:j-1}Z^{n}\right)+\sum_{j\in H_{X|VZ}^{\left(n\right)}}H\left(T\left(j\right)|T^{1:j-1}V^{n}Z^{n}\right)\\ \; & \;\geq |H_{V|Z}^{\left(n\right)}|\left(1-\delta_{n}\right)+|H_{X|VZ}^{\left(n\right)}|\left(1-\delta_{n}\right) \end{array}$$

where we have used the fact that conditioning does not increase entropy, the invertibility of $G_{n}$, and the definition of $H_{V|Z}^{\left(n\right)}$ and $H_{X|VZ}^{\left(n\right)}$ in (6) and (9) respectively. □

Next, the following lemma shows that eavesdropper observations ${\tilde{Z}}_{i}^{n}$ are asymptotically statistically independent of observations ${\tilde{Z}}_{1:i-1}^{n}$ from previous blocks.

**Lemma** **3.** *For any i∈[2,L] and sufficiently large n,*

$$\begin{array}{c} I\left(S_{1:L}{\tilde{Z}}_{1:i-1}^{n};{\tilde{Z}}_{i}^{n}\right)\leq \delta_{n}^{\left(S\right)}, \end{array}$$

*where $\delta_{n}^{\left(S\right)}$ is defined as in Lemma 2.*

**Proof.**  For any i∈[2,L] and sufficiently large *n*, we have

$$\begin{array}{cc} \; & I\left(S_{1:L}{\tilde{Z}}_{1:i-1}^{n};{\tilde{Z}}_{i}^{n}\right)\\ \; & \;=I\left(S_{1:i}{\tilde{Z}}_{1:i-1}^{n};{\tilde{Z}}_{i}^{n}\right)+I\left(S_{i+1:L};{\tilde{Z}}_{i}^{n}|S_{1:i}{\tilde{Z}}_{1:i-1}^{n}\right)\\ \; & \;\overset{\left(a\right)}{=}I\left(S_{1:i}{\tilde{Z}}_{1:i-1}^{n};{\tilde{Z}}_{i}^{n}\right)\\ \; & \;\leq I\left(S_{1:i}{\tilde{Z}}_{1:i-1}^{n}\Xi_{i-1}^{\left(V\right)}\Lambda_{i-1}^{\left(X\right)};{\tilde{Z}}_{i}^{n}\right)\\ \; & \;=I\left(S_{i}\Xi_{i-1}^{\left(V\right)}\Lambda_{i-1}^{\left(X\right)};{\tilde{Z}}_{i}^{n}\right)+I\left(S_{1:i-1}{\tilde{Z}}_{1:i-1}^{n};{\tilde{Z}}_{i}^{n}|S_{i}\Xi_{i-1}^{\left(V\right)}\Lambda_{i-1}^{\left(X\right)}\right)\\ \; & \;\overset{\left(b\right)}{\leq }\delta_{n}^{\left(S\right)}+I\left(S_{1:i-1}{\tilde{Z}}_{1:i-1}^{n};{\tilde{Z}}_{i}^{n}|S_{i}\Xi_{i-1}^{\left(V\right)}\Lambda_{i-1}^{\left(X\right)}\right)\\ \; & \;\leq \delta_{n}^{\left(S\right)}+I\left(S_{1:i-1}{\tilde{Z}}_{1:i-1}^{n};{\tilde{Z}}_{i}^{n}W_{1,i}|S_{i}\Xi_{i-1}^{\left(V\right)}\Lambda_{i-1}^{\left(X\right)}\right)\\ \; & \;=\delta_{n}^{\left(S\right)}+I\left(S_{1:i-1}{\tilde{Z}}_{1:i-1}^{n};W_{1,i}|S_{i}\Xi_{i-1}^{\left(V\right)}\Lambda_{i-1}^{\left(X\right)}\right)+I\left(S_{1:i-1}{\tilde{Z}}_{1:i-1}^{n};{\tilde{Z}}_{i}^{n}|S_{i}\Xi_{i-1}^{\left(V\right)}\Lambda_{i-1}^{\left(X\right)}W_{1,i}\right)\\ \; & \;\overset{\left(c\right)}{=}\delta_{n}^{\left(S\right)}+I\left(S_{1:i-1}{\tilde{Z}}_{1:i-1}^{n};W_{1,i}|S_{i}\Xi_{i-1}^{\left(V\right)}\Lambda_{i-1}^{\left(X\right)}\right)\\ \; & \;\leq \delta_{n}^{\left(S\right)}+I\left({\tilde{A}}_{1:i-1}^{n}{\tilde{Z}}_{1:i-1}^{n};W_{1,i}|S_{i}\Xi_{i-1}^{\left(V\right)}\Lambda_{i-1}^{\left(X\right)}\right)\\ \; & \;=\delta_{n}^{\left(S\right)}+I\left({\tilde{A}}_{1:i-1}^{n};W_{1,i}|S_{i}\Xi_{i-1}^{\left(V\right)}\Lambda_{i-1}^{\left(X\right)}\right)+I\left({\tilde{Z}}_{1:i-1}^{n};W_{1,i}|{\tilde{A}}_{1:i-1}^{n}S_{i}\Xi_{i-1}^{\left(V\right)}\Lambda_{i-1}^{\left(X\right)}\right)\\ \; & \;\overset{\left(d\right)}{=}\delta_{n}^{\left(S\right)}+I\left({\tilde{A}}_{1:i-1}^{n};W_{1,i}|S_{i}\Xi_{i-1}^{\left(V\right)}\Lambda_{i-1}^{\left(X\right)}\right)\\ \; & \;\overset{\left(e\right)}{=}\delta_{n}^{\left(S\right)}+I\left({\tilde{A}}_{1:i-1}^{n};{\bar{\Omega }}_{i}^{\left(V\right)}⊕\kappa_{\Omega }^{\left(V\right)}|S_{i}\Xi_{i-1}^{\left(V\right)}\Lambda_{i-1}^{\left(X\right)}\right)\\ \; & \;\overset{\left(f\right)}{=}\delta_{n}^{\left(S\right)} \end{array}$$

where (a) holds by independence between $S_{i+1:L}$ and any random variable from Blocks 1 to *i*; (b) holds by Lemma 2; (c) follows from applying d-separation [28] over the Bayesian graph in Figure 7 to obtain that ${\tilde{Z}}_{i}^{n}$ and $\left(S_{1:i-1},{\tilde{Z}}_{1:i-1}^{n}\right)$ are conditionally independent given $\left(S_{i},\Xi_{i-1}^{\left(V\right)},\Lambda_{i-1}^{\left(X\right)},W_{1,i}\right)$; (d) also follows from applying *d-separation* to obtain that $W_{1,i}$ and ${\tilde{Z}}_{1:i-1}^{n}$ are conditionally independent given $\left({\tilde{A}}_{1:i-1}^{n},S_{i},\Xi_{i-1}^{\left(V\right)},\Lambda_{i-1}^{\left(X\right)}\right)$; (e) holds by definition; and (f) holds because ${\bar{\Omega }}_{i}^{\left(V\right)}$ is independent of $\left(S_{i},\Xi_{i-1}^{\left(V\right)},\Lambda_{i-1}^{\left(X\right)}\right)$ and any random variable from Block 1 to (i−2), and because from applying crypto-lemma [26] we obtain that ${\bar{\Omega }}_{i}^{\left(V\right)}⊕\kappa_{\Omega }^{\left(V\right)}$ is independent of ${\tilde{A}}_{i-1}^{n}$. □

Therefore, we obtain

$$\begin{array}{cc} I\left(S_{1:L};{\tilde{Z}}_{1:L}^{n}\right)\; & \overset{\left(a\right)}{=}I\left(S_{1:L};{\tilde{Z}}_{1}^{n}\right)+\sum_{i=2}^{L}I\left(S_{1:L};{\tilde{Z}}_{i}^{n}|{\tilde{Z}}_{1:i-1}^{n}\right)\\ \; & \overset{\left(b\right)}{\leq }I\left(S_{1:L};{\tilde{Z}}_{1}^{n}\right)+\left(L-1\right)\delta_{n}^{\left(S\right)}\\ \; & =I\left(S_{1};{\tilde{Z}}_{1}^{n}\right)+I\left(S_{2:L};{\tilde{Z}}_{1}^{n}|S_{1}\right)+\left(L-1\right)\delta_{n}^{\left(S\right)}\\ \; & \overset{\left(c\right)}{=}I\left(S_{1};{\tilde{Z}}_{1}^{n}\right)+\left(L-1\right)\delta_{n}^{\left(S\right)}\\ \; & \overset{\left(d\right)}{\leq }L\delta_{n}^{\left(S\right)} \end{array}$$

where (a) follows from applying the chain rule; (b) holds by Lemma 3; (c) holds by independence between $S_{2:L}$ and any random variable from Block 1; and (d) holds by Lemma 2. Thus, for sufficiently large *n* the polar coding scheme satisfies the strong secrecy condition in (2).

**Remark** **1.** *We conjecture that the use $\kappa_{\Omega }^{\left(V\right)}$ is not needed for the polar coding scheme to satisfy the strong secrecy condition. However, the key is required in order to prove this condition by means of analyzing a causal Bayesian graph similar to the one in Figure 7.*

**Remark** **2.** *Although backward dependencies between random variables of different blocks appear in [12], a secret seed as $\kappa_{\Omega }^{\left(V\right)}$ is not necessary for the polar coding scheme to provide strong secrecy. This is because random sequences that are repeated in adjacent blocks are stored only into those corresponding entries whose indices belong to the “high entropy set given eavesdropper observations”, i.e., the equivalent sets of $H_{V|Z}^{\left(n\right)}$ and $H_{X|VZ}^{\left(n\right)}$ in our polar coding scheme. By contrast, notice that our polar coding scheme repeats $\left[\Theta_{i}^{\left(V\right)},\Gamma_{i}^{\left(V\right)}\right]\subseteq {\tilde{A}}_{i}\left[{\left(H_{V|Z}^{\left(n\right)}\right)}^{C}\right]$.*

**Remark** **3.** *Another possibility for the polar coding scheme is to repeat at Block i+1 the modulo-2 addition between $\left[\Psi_{i}^{\left(V\right)},\Gamma_{i}^{\left(V\right)}\right]$ and a particular secret-key, instead of repeating an* encrypted *version of $\left[\Theta_{i}^{\left(V\right)},\Gamma_{i}^{\left(V\right)}\right]$ at Block i−1. Then, it is not difficult to prove that $I\left(S_{1:L}{\tilde{Z}}_{i+1:L}^{n};{\tilde{Z}}_{i}^{n}\right)\leq \delta_{n}^{\left(S\right)}$ (similar to Lemma 3). Thus, one can minimize the length of this secret-key depending on whether $\left|C_{1}^{\left(n\right)}|<|C_{2}^{\left(n\right)}\right|$ or vice versa.*

## 6. Concluding Remarks

A strongly secure polar coding scheme is proposed for the WTBC with two legitimate receivers and one eavesdropper. This polar code achieves the best known inner-bound on the achievable region of the CI-WTBC model, where a transmitter wants to send common information (private and confidential) to both receivers. Due to the non-degradedness assumption of the channel, the encoder builds a chaining construction that induces bidirectional dependencies between adjacent blocks, which need to be taken carefully into account in the secrecy analysis.

These bidirectional dependencies involve elements from adjacent blocks whose indices belong to the “low entropy sets given eavesdropper observations”. Consequently, in order to prove that the polar coding scheme satisfies the strong secrecy condition, we have introduced a secret-key whose length becomes negligible in terms of rate as the number of blocks grows indefinitely. In the proposed polar coding scheme, this key has been used to randomize part of these elements from any block that are repeated in the previous (or next) one. In this way, we can analyze the dependencies between all random variables involved in the secrecy analysis by means of a causal Bayesian graph and apply d-separation to prove that the polar coding scheme induces eavesdropper’s observations that are statistically independent of one another.

Despite the good performance of the polar coding schemes, some issues still persist. First, it is worth saying that the additional secret transmission (that is negligible in terms of rate) required to initialize the decoding algorithms at both receivers can be omitted by using a similar approach as in [29], where an initialization phase to generate a secret-key can be performed without worsening the communication rate. On the other hand, how to replace the random decisions entirely by deterministic ones in SC encoding is a problem that still remains unsolved. Additionally, we conjecture that the previous secret-keys that are used to prove independence between blocks are not necessary. However, how to prove this independence without using them seems a difficult problem to address at this point.

## Abbreviations

The following abbreviations are used in this manuscript:

WTBC	Wiretap broadcast channel
CI-WTBC	Common information over the wiretap broadcast channel
SC	Successive cancellation
DMS	Discrete memoryless source
